# Ethnic group inequalities in coverage with reproductive, maternal and child health interventions: cross-sectional analyses of national surveys in 16 Latin American and Caribbean countries

**DOI:** 10.1016/S2214-109X(18)30300-0

**Published:** 2018-07-14

**Authors:** Marilia Arndt Mesenburg, Maria Clara Restrepo-Mendez, Hugo Amigo, Alejandra D Balandrán, Maria Angelica Barbosa-Verdun, Beatriz Caicedo-Velásquez, Liliana Carvajal-Aguirre, Carlos E A Coimbra, Leonardo Z Ferreira, Maria del Pilar Flores-Quispe, Carlos Flores-Ramírez, Giovanna Gatica-Dominguez, Luis Huicho, Karla Jinesta-Campos, Ingrid S K Krishnadath, Fatima S Maia, Ivan A Marquez-Callisaya, Mercedes Marlene Martinez, Oscar J Mujica, Verónica Pingray, Alejandro Retamoso, Paulina Ríos-Quituizaca, Joel Velásquez-Rivas, Carlos A Viáfara-López, Sasha Walrond, Fernando C Wehrmeister, Fabiana Del Popolo, Aluisio J Barros, Cesar G Victora

**Affiliations:** a International Center for Equity in Health, Federal University of Pelotas, Pelotas, Brazil; b Postgraduate Program in Epidemiology, Federal University of Pelotas, Pelotas, Brazil; c MRC Integrative Epidemiology Unit, Population Health Sciences, University of Bristol, Bristol, UK; d Nutrition Department, Universidad de Chile, Santiago, Chile; e Secretariat of Health, Mexico City, Mexico; f Department of Epidemiology, Hospital Materno Infantil San Pablo, Asuncion, Paraguay; g Epidemiología, Facultad Nacional de Salud Pública, Universidad de Antioquia, Medellín, Colombia; h United Nations Children's Fund, New York City, NY, USA; i Escola Nacional de Saúde Pública Sérgio Arouca, Fundação Oswaldo Cruz, Brazil; j Ministry of Public Health and Social Assistance, Ciudad de Guatemala, Guatemala; k Centro de Investigación para el Desarrollo Integral y Sostenible, Centro de Investigación en Salud Materna e Infantil and School of Medicine, Universidad Peruana Cayetano Heredia, Lima, Peru; l Universidad Nacional Mayor de San Marcos, Lima, Peru; m Instituto Nacional de Estadística y Censos, San José, Costa Rica; n Department of Public Health, Faculty of Medical Sciences, Anton de Kom University of Suriname, Paramaribo, Suriname; o Department of Public Health, Faculty of Medical Science, Federal University of Rio Grande, Rio Grande, Brazil; p Instituto Nacional de Estadística, La Paz, Bolivia; q Posgrado en Salud Pública/FCM, Universidad Nacional Autónoma de Honduras, Tegucigalpa, Honduras; r Pan American Health Organization, Washington, DC, USA; s Institute for Clinical Effectiveness and Health Policy, Buenos Aires, Argentina; t United Nations Children's Fund, Montevideo, Uruguay; u FCM, Universidad Central del Ecuador, FMRP, Universidad Sao Paulo, Quito, Ecuador; v Ministry of Health, Managua, Nicaragua; w Departament de Economía Universidad del Valle, Cali, Colombia; x Ministry of Public Health, Georgetown, Guyana; y Comisión Económica para América Latina y el Caribe, Santiago, Chile

## Abstract

**Background:**

Latin American and Caribbean populations include three main ethnic groups: indigenous people, people of African descent, and people of European descent. We investigated ethnic inequalities among these groups in population coverage with reproductive, maternal, newborn, and child health interventions.

**Methods:**

We analysed 16 standardised, nationally representative surveys carried out from 2004 to 2015 in Latin America and the Caribbean that provided information on ethnicity or a proxy indicator (household language or skin colour) and on coverage of reproductive, maternal, newborn, and child health interventions. We selected four outcomes: coverage with modern contraception, antenatal care coverage (defined as four or more antenatal visits), and skilled attendants at birth for women aged 15–49 years; and coverage with three doses of diphtheria-pertussis-tetanus (DPT3) vaccine among children aged 12–23 months. We classified women and children as indigenous, of African descent, or other ancestry (reference group) on the basis of their self-reported ethnicity or language. Mediating variables included wealth quintiles (based on household asset indices), woman's education, and urban-rural residence. We calculated crude and adjusted coverage ratios using Poisson regression.

**Findings:**

Ethnic gaps in coverage varied substantially from country to country. In most countries, coverage with modern contraception (median coverage ratio 0·82, IQR 0·66–0·92), antenatal care (0·86, 0·75–0·94), and skilled birth attendants (0·75, 0·68–0·92) was lower among indigenous women than in the reference group. Only three countries (Nicaragua, Panama, and Paraguay) showed significant gaps in DPT3 coverage between the indigenous and the reference groups. The differences were attenuated but persisted after adjustment for wealth, education, and residence. Women and children of African descent showed similar coverage to the reference group in most countries.

**Interpretation:**

The lower coverage levels for indigenous women are pervasive, and cannot be explained solely by differences in wealth, education, or residence. Interventions delivered at community level—such as vaccines—show less inequality than those requiring access to services, such as birth attendance. Regular monitoring of ethnic inequalities is essential to evaluate existing initiatives aimed at the inclusion of minorities and to plan effective multisectoral policies and programmes.

**Funding:**

The Bill & Melinda Gates Foundation (through the Countdown to 2030 initiative) and the Wellcome Trust.

## Introduction

In contrast to the Millennium Development Goals (2000–15), which relied upon national-level statistics for assessing country progress, the Sustainable Development Goals (2015–30) call for the production of statistics that are “disaggregated by income, gender, age, race, ethnicity, migratory status, disability, geographic location and other characteristics relevant in national contexts”.^1^ Among the dimensions of inequality, ethnicity is particularly difficult to measure in a consistent or standardised way across countries because it is a social construct that comprises several aspects of identity such as ancestry, appearance, cultural customs, place of birth, and shared history that are generally not directly measured in epidemiological surveys.^2^, ^3^ It is not surprising, therefore, that there are few multicountry studies of ethnic inequalities compared with the large number of analyses focusing on inequalities associated with wealth, education, age, or place of residence.

**Research in context**

**Evidence before this study**

We searched PubMed, Popline, Lilacs, Portal Capes, and Web of Science for combinations of regional keywords (Latin America or Central America or South America or Caribbean) with the terms “ethnic disparities”, “indigenous health” or “health, indians” with no language restrictions. We also searched the grey literature, particularly the websites of the Latin American Economic Commission, the Latin American Demography Center, the Inter-American Development Bank, UNICEF, Minority Rights Group International and the United Nations. All searches were for content published up to June 30, 2017. We extracted articles and reports that provided results for more than one country.

The multicountry literature on ethnicity and health is mostly focused on mortality, fertility, and nutrition outcomes. In an extensive review of 28 indigenous populations in 23 countries, Anderson and colleagues showed that child undernutrition, maternal mortality, and infant mortality are consistently more common among indigenous than non-indigenous populations. Six Latin American countries were included in this review, which did not include analyses of coverage with reproductive, maternal, newborn, and child health (RMNCH) interventions. Two other reports from the Latin America and the Caribbean Demographic Centre showed consistent coverage gaps for antenatal, delivery, and postnatal care for indigenous women compared with the rest of the population in up to seven countries. In Mexico and Peru, there is evidence that the gap in coverage with skilled birth attendants between indigenous and non-indigenous women is narrowing over time. A separate publication showed lower contraceptive coverage for indigenous compared with non-indigenous women in eight countries. Higher levels of under-5 mortality for indigenous children were also reported in a study involving 11 countries in the region, even when urban-rural residence status is adjusted for.

In spite of the well recognised social disadvantages faced by people of African descent in Latin America, information on health indicators for this group is even scarcer than that available for indigenous women and children. One study showed that children of African descent are at higher risk of infant mortality than are a reference category that did not include indigenous children, but multicountry studies on intervention coverage are not available.

**Added value of this study**

To our knowledge, this study is the largest multicountry analysis on how coverage with health services varies according to the three main ethnic groups in Latin America and the Caribbean. We are unaware of any previous analysis including both indigenous women and children and those of African descent. We use data from 16 national surveys to provide comprehensive analyses of ethnic group differences in RMNCH coverage in the region. We also assess whether socioeconomic differences account for the observed disparities among ethnic groups.

**Implications of all the available evidence**

On the basis of the coverage of four health interventions that have been available for decades, we show significant gaps between indigenous women and children and the rest of the population. People of African descent, for the most part, showed similar coverage levels to those in the reference group. Our analyses revealed several shortcomings in how ethnicity is measured in existing surveys. Better measurement is needed to monitor trends in inequalities, assess the impact of inclusive policies that are being pursued by several countries, and help to design effective policies and programmes.

The multicountry literature on ethnicity and health is mostly focused on mortality, fertility, and nutrition outcomes^2^, ^4^ rather than on coverage with reproductive, maternal, newborn, and child health (RMNCH) interventions. Intervention coverage is defined as the proportion of the population in need of a service or a biologically active product that actually receives them.^5^ Examples include family planning, antenatal care, and delivery care, as well as preventive and curative interventions delivered to children such as vaccines, micronutrients, and antimicrobials. Publications on ethnic disparities in RMNCH in Latin America and the Caribbean cover up to 11 countries,^6^, ^7^, ^8^, ^9^ showing important gaps between indigenous and non-indigenous women and children. In these analyses, infant mortality levels around the year 2010 were between 31% and 220% higher among indigenous children compared with non-indigenous children except for in Costa Rica, where the difference was only 2%.^10^ The literature on health indicators for people of African descent is even more scarce.^7^, ^8^, ^11^, ^12^

The present population of countries in the Latin American and Caribbean region includes three major ethnic groups—namely, indigenous people, people of African descent, and people of European descent—except in Guyana, Suriname, and some Caribbean countries. The history of conquest and slavery in this region has resulted in ethnically and economically stratified societies despite intense miscegenation. Evidence from economic and demographic surveys suggests that important ethnic inequalities continue to persist.^4^, ^13^, ^14^

Countdown to 2030 is a global initiative aimed at tracking country-level progress in the coverage of essential RMNCH interventions.^15^ Countdown promotes collaboration among country-level academics and policy makers in producing analyses of population coverage with essential health interventions, with particular emphasis on documentation of within-country inequalities. We are not aware of any multicountry analyses on RMNCH coverage by ethnic groups that have made full use of recently available survey data, nor of any analyses addressing whether socioeconomic differences can account for the ethnic gaps in coverage of health interventions.

We present a set of analyses of ethnic inequalities in RMNCH coverage that involved the effort of researchers from 16 countries in the region of Latin America and the Caribbean, resulting from two workshops sponsored by the Countdown to 2030.

## Methods

### Data sources

We analysed standardised, nationally representative surveys carried out in Latin America and the Caribbean that provided information on ethnicity or a proxy indicator (ie, language or skin colour) and on coverage with RMNCH interventions. We included surveys for which datasets were publicly available from Jan 1, 2000, to June 30, 2017.

Three international survey initiatives—Demographic and Health Surveys (DHS), Multiple Indicator Cluster Surveys (MICS), and Reproductive and Health Surveys (RHS)—have been active in the region since 1985. The three survey initiatives use similar questionnaires and their results are frequently pooled for global and regional analyses.^16^, ^17^ All surveys employ multistage sampling procedures to select women aged 15–49 years for interview; more details on their methodologies are available elsewhere.^18^, ^19^, ^20^ A review in July, 2017, of the websites of the survey initiatives was complemented by contacts with key informants from countries and international organisations in the region to ensure that all surveys that had been carried out since 2000 were identified.

We identified 16 surveys, each pertaining to a distinct country, with information on ethnicity and information on at least one of the four indicators analysed (appendix): five DHS (Bolivia, 2008; Colombia, 2010; Guatemala, 2014; Honduras, 2011; and Peru, 2012), seven MICS (Belize, 2011; Costa Rica, 2011; Guyana, 2014; Mexico, 2015; Panama, 2013; Suriname, 2010; and Uruguay, 2012), three RHS (Ecuador, 2004; Nicaragua, 2006; and Paraguay 2008), and a modified version of DHS in Brazil (2006) conducted by the Brazilian Center for Analysis and Planning, which was fully based on the DHS but included some additional topics of national interest. The references to the final reports of the surveys included in the analyses are presented in the appendix. Some countries in the region have had more recent surveys than those included in our analysis, but their datasets did not provide standardised indicator definitions or were not available to the research team at the time of the analyses. For countries with more than one survey, we included the most recent one.

Ethical approval was obtained by the national agencies responsible for each survey. All analyses relied on publicly available, anonymised databases.

### Coverage indicators

The population of interest for the analyses were women aged 15–49 years and children aged 12–23 months for whom data were available in the included surveys. On the basis of data availability and the need to include RMNCH interventions along the continuum of care, we selected four outcomes: coverage with modern contraception, antenatal care coverage, skilled birth attendant coverage, and diphtheria, tetanus, and pertussis vaccine (DPT3) coverage.

As an indicator of coverage with modern contraception, we considered women (married or in union) who were using, or whose partners were using, a modern contraceptive at the time of the survey; modern methods include oral contraceptives (referred to in the surveys as the pill), condoms (male and female), intrauterine devices, sterilisation (male and female), injectables, implants (eg, Norplant), diaphragm, spermicidal agents (foam or jelly), patches, and emergency contraception (the day-after pill).

As an indicator of antenatal care coverage, we considered women who had delivered a child in the recent past (3 years before the survey for DHS and RHS and 2 years before for MICS) who reported having attended four or more antenatal visits during their last pregnancy. For this same group of women, we considered those who reported that the most recent delivery had been assisted by a skilled health worker (eg, a doctor, nurse, or midwife) to be an indicator of skilled birth attendant coverage.

As an indicator of DPT3 coverage, we considered children aged 12–23 months who had received three doses of DPT3 or another vaccine (tetravalent or pentavalent vaccines) that contained the DPT antigens.

After downloading the survey databases, we calculated the coverage indicators using the Countdown to 2030 definitions^21^ for the numerators, denominators, and age ranges. All analyses were carried out at the individual level within each survey. Missing values were treated as lack of coverage; see the appendix for the frequency of missing values for each indicator. Of the 61 analyses, only two variables had more than 10% of missing values: antenatal care in Belize and in Suriname.

### Stratification variables

For inclusion in the analyses, surveys had to provide information on at least one of ethnicity, language, or self-reported skin colour (see table for the variables available in each country and their respective categories); we chose these variables because data on them are typically collected in censuses and nationally representative surveys. In Brazil, Ecuador, and Uruguay, the questions that sourced this information varied slightly, but the answers included a mixture of self-reported skin colours and ethnicity, with the categories such as white, mestizo, brown, black, yellow (for Asians), and indigenous (table).

**Table:** Data sources and definitions of ethnicity for the countries included in the analyses

	**Definition (as given in original questionnaire)**
**Belize, 2011 (MICS): ethnicity (head of household)**	
Reference	Mestizo, others
Indigenous	Maya
African descent	Creole, Garifuna
**Bolivia, 2008 (DHS): ethnicity (woman)**	
Reference	No ethnic affiliation declared
Indigenous	Quechua, Aymara, Guarani, other indigenous group
African descent	Not available
**Brazil, 2006 (modified DHS): ethnicity or skin colour (woman)**	
Reference	White
Indigenous	Indigenous
African descent	Brown or black
**Colombia, 2010 (DHS): ethnicity (woman)**	
Reference	Other
Indigenous	Native Colombian
African descent	Raizal from archipelago, Palanquero from San Basilio, black/mulato/Afro-Colombian/Afro-descendant
**Ecuador, 2004 (RHS): ethnicity or skin colour (woman)**	
Reference	White, mestizo
Indigenous	Indigenous
African descent	Black
**Costa Rica, 2011 (MICS): ethnicity (head of household)**	
Reference	Others
Indigenous	Indigenous
Afrodescendant	Black/Afrocostarricense
**Guatemala, 2014 (DHS): ethnicity (woman)**	
Reference	Ladino/mestizo
Indigenous	Maya, Xinca
African descent	Not available
**Guyana, 2014 (MICS): ethnicity (head of household)**	
Reference	Mixed race/east Indian
Indigenous	Amerindian
African descent	African
**Honduras, 2011 (DHS): ethnicity (woman)**	
Reference	No ethnic affiliation declared
Indigenous	Tolupan, Pech (Paya), Misquito, Nahoa, Lenca, Tawaka (Sumo), Maya chorti
African descent	Garifuna, black English
**Mexico, 2015 (MICS): ethnicity (head of household)**	
Reference	Non-indigenous household
Indigenous	Indigenous household
African descent	Not available
**Nicaragua, 2006 (RHS): ethnicity (woman)**	
Reference	Mestizo from the Caribbean coast, no ethnicity declared
Indigenous	Rama, Mayangna-Sumu, Miskitu, Ulwa, Xiu-Sutiava, Nahoa-Nicarao, Chorotega-Nahua-Mange, Cacaopera-Matagalpa
African descent	Not available
**Panama, 2013 (MICS): ethnicity (head of household)**	
Reference	Others
Indigenous	Indigenous
African descent	Black or afro-descendant
**Paraguay, 2008 (RHS): language (household)**	
Reference	Spanish or Spanish/Guarani
Indigenous	Guarani-only
African descent	Not available
**Peru, 2012 (DHS): language (household)**	
Reference	Spanish
Indigenous	Quechua, Aymara, other indigenous
African descent	Not available
**Suriname, 2010 (MICS): ethnicity (head of household)**	
Reference	Creole/Indian/Javanese/mixed race
Indigenous	Indigenous/Amerindian
African descent	Marron
**Uruguay, 2012 (MICS): ethnicity or skin colour (head of household)**	
Reference	White or other
Indigenous	Indigenous
African descent	Afrodescendant or black

MICS=Multiple Indicator Cluster Surveys. DHS=Demographic and Health Surveys. RHS=Reproductive and Health Surveys.

We used three broad categories in the analyses, customised at national level: indigenous people, people of African descent, and the reference group, which included people of European descent and those of mixed ancestry in most countries. We chose this reference group because these populations are typically privileged in terms of socioeconomic and health indicators owing to their national histories of colonialism and immigration. Suriname and Guyana had special classifications for the reference group (table; appendix p 7).

A wealth index was calculated for each survey through principal component analyses of household assets and building characteristics. The first component resulting from the analyses was divided into quintiles, with Q1 representing the poorest and Q5 the wealthiest 20% of all households.^22^, ^23^, ^24^ In each of the surveys, maternal education was recorded as none, any primary education, and any secondary education or more, and urban or rural residence was used as defined by the local census bureaus.

### Statistical analysis

All data were harmonised by the analytical team, who for more than a decade have produced the analyses of inequalities in RMNCH^25^ for the Countdown to 2030 and the Health Equity Monitor of WHO.

Initial analyses included the cross tabulation of ethnicity against the four coverage outcomes. Coverage ratios were calculated for indigenous people and people of African descent separately compared with the reference categories, using Poisson regression for outcomes coded as binary variables. This approach has the advantage of providing results as prevalence ratios, which are easier to communicate to non-specialists than are odds ratios resulting from logistic regression. The robust variance option for Poisson regression ensures the assumptions behind the regression model are not violated.^26^ Coupled with the robust variance option or the “svy” prefix within Stata for survey sample estimation, the Poisson regression SEs are estimated taking into account observed data variation in such a way that distributional assumptions of the model are not violated. The multistage sampling, sample weights, and the clustered nature of the data were accounted for using the “svy” series of commands in Stata 13 (StataCorp, College Station, TX, USA).

We used crude models to assess how much coverage varied by ethnicity. We used the adjusted models to investigate whether any coverage gaps could be explained by differences between the ethnic groups in terms of household wealth, education of the woman, or urban-rural residence. Adjusted coverage ratios were calculated using Poisson regression. To decide whether pooling the data from the 16 countries was appropriate, we carried out random-effects meta-analyses and meta-regression procedures, including the following covariates: year of the survey, gross national product per capita,^27^ and national percentages of urbanisation, indigenous people, and those of African descent; the latter three variables were obtained from the surveys under analysis. We tested for heterogeneity among country results using the *I*^2^ statistic.^28^

### Role of the funding source

The funding sources did not have any role in the design, conduct, analysis, or writing up of the study. The corresponding author had full access to all the study data and had final responsibility for the decision to submit for publication.

## Results

Data were available for 16 countries, 14 of which had information on all four coverage outcomes (data were only available on antenatal and delivery care for Uruguay and information on DPT3 vaccination was not available for Brazil). 12 surveys provided information on self-reported ethnic affiliation, two on skin colour, and two on the language spoken at home (table). Nine countries (Belize, Brazil, Colombia, Costa Rica, Ecuador, Guyana, Honduras, Panama and Suriname) had information on three ethnic categories (indigenous people, people of African descent, and the reference group), six countries (Bolivia, Guatemala, Mexico, Nicaragua, Paraguay, and Peru) had information on indigenous and reference groups, and Uruguay had results for people of African descent and the reference group. Categories with fewer than 25 unweighted observations were excluded from the analyses: people of African descent in Costa Rica (DPT3 indicator) and in Nicaragua (all indicators) and indigenous people in Uruguay (all indicators).

Taken together, the 16 surveys included 132 823 women and 19 313 children aged 12–23 months. The numbers of women and children used in the calculation of each indicator are shown in the appendix. The median number of clusters in the national samples was 591 (IQR 368–1000), ranging from 196 in Belize to 3984 in Colombia.

**Figure 1:**
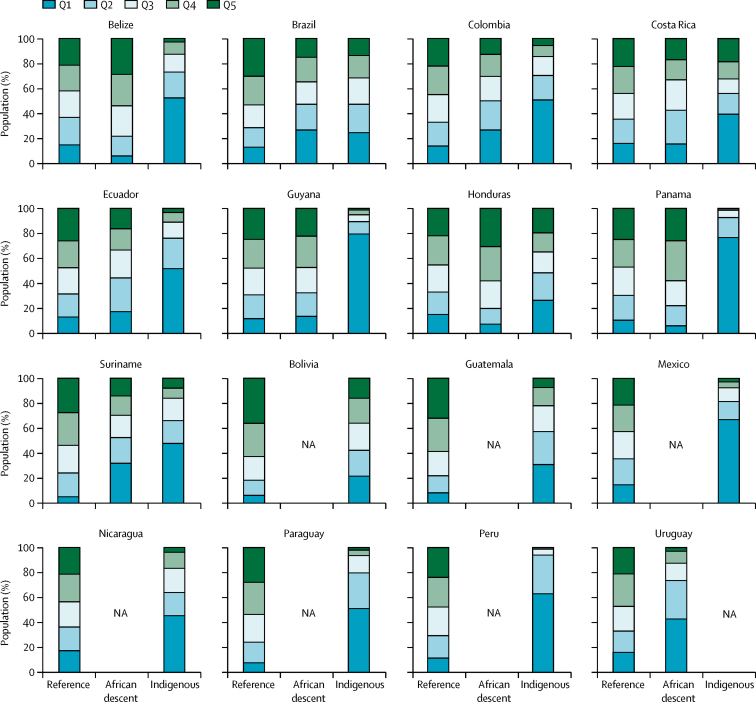
Distribution of the three ethnic groups according to wealth quintiles, by country Q1 is the poorest quintile and Q5 is the wealthiest quintile. NA=not available.

Compared with people of African descent and with the reference group, indigenous women were more likely to belong to the poorest wealth quintiles, have less years of formal education, and live in rural areas in almost all countries (figure 1; appendix). More than 50% of indigenous women were in the poorest quintile in eight of the 15 countries with available data (figure 1). Socioeconomic differences between people of African descent and the reference group in terms of wealth and education were less marked, except in Brazil, Colombia, Suriname, and Uruguay. Except in Guyana, a higher proportion of people of African descent were urban than were rural (appendix).

Coverage levels for each country for the four outcomes under study are presented in the appendix. Median coverage levels in the reference group were 64% (IQR 54–71) for modern contraception, 89% (86–93) for antenatal care, 96% (88–98) for skilled birth attendants, and 87% (75–93) for DPT3 vaccine (appendix). Similar median values were observed for people of African descent (64% [IQR 53–68], 87% [78–94], 97% [90–99], and 81% [74–91], respectively), whereas those for indigenous women and children were substantially lower (44% [31–61], 74% [61–84], 73% [62–91], and 81% [73–88], respectively).

**Figure 2:**
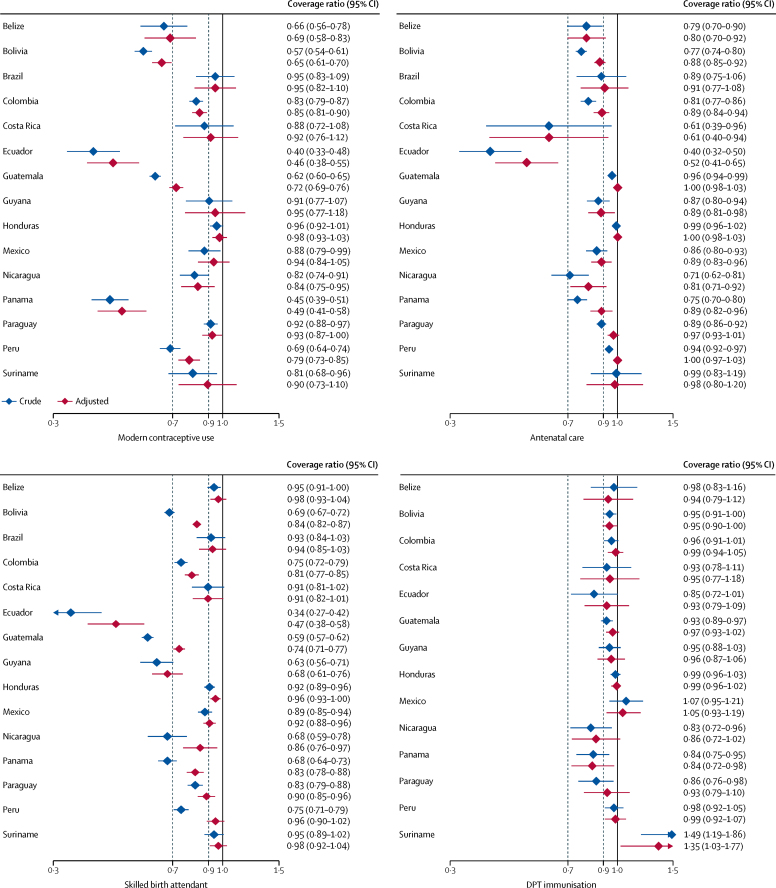
Crude and adjusted coverage ratios for RMNCH interventions in indigenous women and children compared with the reference category, by country The dashed lines show coverage ratios below 0·9 and 0·7, which were chosen to represent low and very low coverage ratios, respectively. RMNCH=reproductive, maternal, newborn, and child health. DPT=diphtheria-pertussis-tetanus.

In 11 of the 15 countries with information on modern contraceptive use, coverage was significantly lower in the indigenous population than in the reference group in the unadjusted analyses (figure 2; appendix). In most countries, adjustment for wealth, education, and urban-rural residence attenuated the differences, but these were still significant in eight countries: Belize, Bolivia, Colombia, Ecuador, Guatemala, Nicaragua, Panama, and Peru. The median values for the contraceptive coverage ratios were 0·82 (IQR 0·66–0·92) in the crude analyses and 0·85 (0·69–0·94) in the adjusted analyses.

12 of the 15 countries with information on antenatal care showed unadjusted coverage ratios that were significantly lower in the indigenous population than in the reference group (figure 2; appendix). After adjustment, significant differences were still present in Belize, Bolivia, Colombia, Costa Rica, Ecuador, Guyana, Mexico, Nicaragua, and Panama (figure 2). The median values for the coverage ratios were 0·86 (IQR 0·75–0·94) in the crude analyses and 0·89 (0·81–0·98) in the adjusted analyses.

Data on skilled birth attendants were available for 15 countries, of which 11 showed unadjusted coverage ratios significantly lower in indigenous populations than in the reference group (figure 2; appendix). After adjustment, coverage remained lower in nine countries: Bolivia, Colombia, Ecuador, Guatemala, Guyana, Mexico, Nicaragua, Panama, and Paraguay. The median values for the coverage ratios were 0·75 (IQR 0·68–0·92) in the crude analyses and 0·90 (0·81–0·96) in the adjusted analyses.

Four of the 14 countries with information on DPT3 immunisation had coverage ratios significantly lower for indigenous children compared with the reference group (figure 2; appendix). In Suriname, coverage was 49% higher for indigenous children than for those belonging to the reference group; coverage in the reference group was only 51%, the lowest in the region (appendix). Indigenous children from Panama still had lower coverage after adjustment, whereas in Suriname they presented higher coverage levels in both crude and adjusted analyses (figure 2). The median values for DPT3 coverage ratios were 0·95 (IQR 0·86–0·98) in the crude analyses and 0·96 (0·93–1·05) in the adjusted analyses.

We tested for heterogeneity among countries, as a preliminary step for using a random-effects meta-analytic procedure to obtain pooled results. The *I*^2^ statistic for heterogeneity was 97·2% for contraception, 94·4% for antenatal care, 97·3% for skilled birth attendants, and 65·1% for DPT3 vaccination, indicating high heterogeneity and suggesting that pooling was not recommended. The meta-regression procedure (appendix) showed that the covariates were unable to explain the heterogeneity, except possibly for antenatal care for indigenous women (*R*^2^ 46·1%) and skilled attendance at delivery for women of African descent (26·8%).

**Figure 3:**
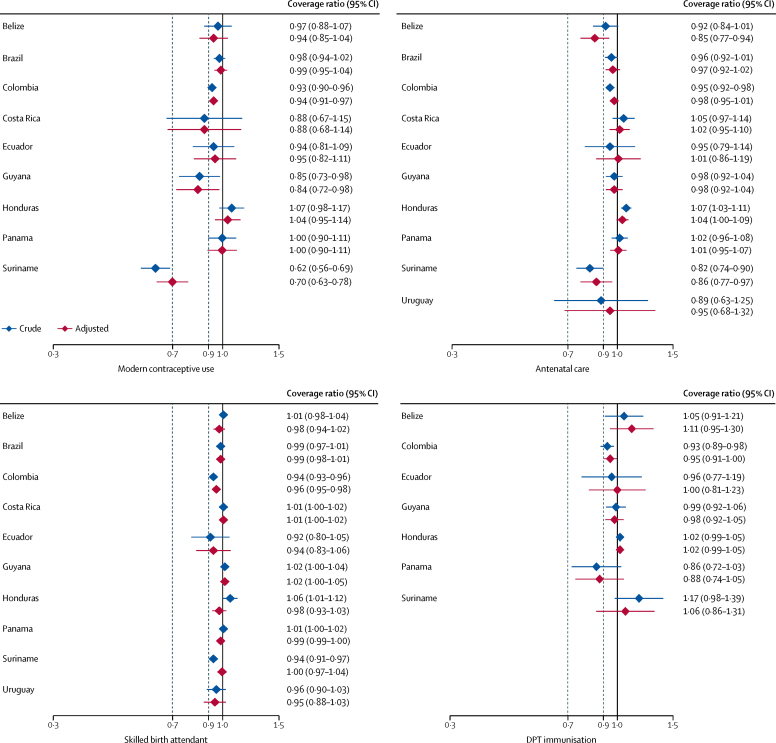
Crude and adjusted coverage ratios for RMNCH interventions in women and children of African descent compared with the reference category, by country The dashed lines show coverage ratios below 0·9 and 0·7, which were chosen to represent low and very low coverage ratios, respectively. RMNCH=reproductive, maternal, newborn, and child health. DPT=diphtheria-pertussis-tetanus.

When considering the nine countries with information on modern contraception, the unadjusted ratios were significantly lower in women of African descent compared with the reference group in Suriname, Guyana, and Colombia (figure 3; appendix). After adjustment, the ratios in these three countries remained significant. Unlike what was observed for indigenous women, adjustment for wealth, education, and residence had small and inconsistent effects on the estimates. The median values for contraceptive coverage ratios were 0·94 in both the crude and adjusted analyses (with IQRs 0·88–0·98 for the crude analysis and 0·88–0·99 for the adjusted analysis).

Women of African descent had similar levels of antenatal care coverage to the reference group in most of the nine countries with available information; coverage for women of African descent was lower than for the reference group in Colombia and Suriname and higher in Honduras (figure 3). After adjustment, the ratios remained significant only in Suriname, and in Belize adjustment accentuated the ratio and coverage among women of African descent became significantly lower than in the reference group (figure 3). The median values for antenatal coverage ratios were 0·96 (IQR 0·92–1·02) in the crude analyses and 0·98 (0·95–1·01) in the adjusted analyses.

Women of African descent had similar skilled birth attendant coverage to the reference group in nearly all countries (figure 3). In Colombia and Suriname, coverage was lower in the crude analyses, but after adjustment only Colombia remained significant, with a small difference in coverage. The median values for coverage ratios were 1·00 (IQR 0·94–1·01) in the crude analyses and 0·99 (0·96–1·00) in the adjusted analyses.

DPT3 coverage data were available for seven countries. Only the ratio for Colombia was significantly lower in the crude analyses but not after adjustment, with none of the countries showing a difference between people of African descent and the reference group. The median values for DPT3 coverage ratios were 0·99 (IQR 0·93–1·05) in the crude analyses and 1·00 (0·95–1·06) in the adjusted analyses.

Similar to the analyses of coverage among indigenous groups, the *I*^2^ statistic showed great heterogeneity across countries: 89·7% for contraception, 79·9% for antenatal care, 90·2% for skilled attendance, and 64·4% for DPT3 vaccination, again suggesting that pooling results from different countries was not advisable.

## Discussion

To our knowledge, this is the largest systematic study of ethnic group inequalities in RMNCH coverage in the Americas, covering 16 countries that account for 78·5% of all women aged 15–49 years in the region.^29^ We attempted to identify all surveys carried out in the region since 2000 that had information on ethnicity and intervention coverage. Our analyses are hampered by the limitations of survey data in terms of ascertaining ethnicity. We relied on three self-reported variables as proxies of ethnic group affiliation: declared ethnicity, language spoken at home, and skin colour. The reference groups varied from country to country, and included either women who did not declare themselves as indigenous or of African descent, those who reported speaking Spanish at home, and those who considered themselves as being white. Because miscegenation is widespread in the region, the reference category for the analyses includes many women and children with mixed European, indigenous, and African ancestries^30^ (also see appendix for details on ethnic groups in Suriname and Guyana, where the ethnic groups differ from those present in most of the region). Our reference group, therefore, encompasses individuals with a wide range of socioeconomic and cultural characteristics.^4^ It is likely that, had it been possible to identify a reference group with stronger European ancestry, the observed ethnic gaps would have been even wider.

A related issue is that most surveys included a single option for people of African descent, but in Brazil information on “brown” and “black” skin colours was collected separately, and both groups were pooled in our analyses for consistency with the other surveys. When analysed separately, there were increasing gradients in coverage for women who self-reported as black, brown, and white (appendix), consistently with the Brazilian literature on maternal and child health outcomes.^31^, ^32^ It should be noted that ethnic classification according to self-reported skin colour has been officially adopted in Brazil and is supported by the organised Black Movement that, since the 1970s, has advocated for disaggregation of all vital and health statistics according to skin colour.^33^

Given the complexity of ethnic group classifications,^2^ the proportions of the survey samples classified in each group might differ from those measured in population censuses or in other surveys. An example is Costa Rica, where, according to the 2011 MICS, people of African descent represented 4·7% and indigenous people 2·4% of all women with children, whereas the 2011 national census showed the proportions to be 2·5% and 1·1%, respectively. Such discrepancies might arise from the types of questions and categories used in each source and, in the case of sample surveys, the small number of observations when the information is disaggregated into subgroups.^14^ Therefore, our results must be interpreted with due caution.

The appendix provides detailed information from the 16 countries showing that the number of indigenous groups recognised by the Census Offices ranges from one in Uruguay, Guyana, and Suriname to more than 300 in Brazil. Therefore, using a single category of “indigenous” to group such a broad variety of nations, each with unique languages, customs, and traditional healing systems outside of the prevailing health-care system, is an important limitation of our present analyses. However, given issues of comparability, data availability, and sample size limitations, we had no other alternative than to present pooled results for all indigenous groups. To some extent, this also applies to people of African descent, who in some countries bring together many separate and rather different cultures. It is also important to note that the legal status of indigenous populations, and the government institutions in charge of their affairs, varies markedly from country to country (appendix).

Although the survey sampling schemes did not allow for ethnicity-specific sampling domains, the numbers of clusters studied are large with a median of 591 (IQR 368–1000) per survey. Analyses of wealth quintiles and women's education, for example, are commonly done in such surveys even though sampling schemes are not specifically designed with these stratification variables in mind.

Another limitation to our study is the absence of certain categories—eg, surprisingly the Peruvian survey did not collect information on people of African descent, who represent 5–9%^34^, ^35^ of the country's population—and the small sample sizes for some categories (eg, the indigenous population in Uruguay and Brazil). Sample sizes might be affected by the concentration of some ethnic groups in specific geographical areas from which few clusters were selected through random sampling. We hope that future studies will take the importance of ethnicity into account and will collect detailed information and oversample areas where minorities are concentrated.

Our results span over 11 years—from 2004 to 2015—owing to the difference in the dates of the available surveys, and ethnic self-identification can change over time. Nevertheless, the year of the survey did not explain the heterogeneity among countries (appendix). In countries with older surveys such as Ecuador, the current magnitude of inequalities might be different from that reported here. Lastly, our analyses do not take into account traditional healing systems used by indigenous populations and people of African descent, but are focused on evidence-based interventions typical of high-income countries.^36^

The conceptual model behind our analyses proposed two levels of determination of health outcomes.^37^ Ethnicity represents a distal or structural determinant, whereas wealth, education, and urban-rural residence are proximate determinants or mediators. The unadjusted models show the full effect of ethnicity, whereas the adjusted models show how much of an effect remains after considering socioeconomic position and urbanisation.^37^ Our results show that, in most countries, indigenous women and children were markedly poorer than the reference group, confirming several reports from Latin America and the Caribbean.^4^, ^13^, ^34^, ^35^, ^38^ In some, but not all countries, people of African descent were poorer than the reference group, and in all countries—except for Brazil—people of African descent were wealthier than indigenous populations.

Our analyses on intervention coverage show that indigenous women were systematically excluded from the three reproductive and maternal interventions under study. When considering the point estimates alone, in 14 of the 42 crude analyses (ie, analyses of the three indicators with data available in 14 countries), indigenous women had coverage figures below 70% of the coverage attained in the reference category, and in another 17 analyses, their coverage was between 70% and 89% of that in the reference group. Among the countries studied, the widest gaps were seen in Ecuador in 2004, where the three ratios were under 0·5, followed by Bolivia, Guatemala and Panama, where, considering the point estimates alone, two of the three ratios were under 0·7. Honduras and Suriname were the best performers. Results from Ecuador confirm the existence of marked ethnic gaps in the country,^39^ but these could have changed since the 2004 survey. Among the interventions delivered to women, antenatal care was less inequitable than either contraceptive or skilled attendant coverage. Antenatal care attendance is a requirement or conditionality for several cash-transfer programmes implemented in the region.^40^ Our results are consistent with the findings of earlier analyses based on a smaller number of countries and using a more restricted set of outcome variables.^6^, ^7^, ^8^, ^9^

By contrast with interventions delivered to women, vaccination coverage was quite equitable in most countries. Suriname showed higher coverage among indigenous people than among the reference group. Earlier analyses of wealth-related inequalities in coverage in Countdown to 2030 countries showed that vaccines—often delivered at community level through campaigns—are more equitably distributed than are interventions that require access to health facilities, which might incur additional costs in user fees and transportation.^41^

Adjusted analyses showed that, in most cases, the lower coverage among indigenous women was somewhat attenuated when poverty, fewer years of formal education, and rural residence were accounted for. Nonetheless, important inequalities persisted, suggesting that other mediating factors are present or discrimination or institutional barriers might play a part.^4^, ^6^, ^7^, ^13^ An analysis of the intercultural health programmes introduced in several countries in the region is beyond the scope of the present Article, but systematic collection of standardised survey data over time is essential to assess the impact of such programmes.^7^

It is important to recognise the wide heterogeneity of our results, which prevented us from carrying out meta-analyses. This finding reinforces the need to consider national contexts when studying ethnic inequalities. For example, in four countries (Brazil, Honduras, Mexico, and Suriname), the gaps between indigenous populations and the reference category were small, with the average coverage gap for the four interventions being 10% or less. Policy and programmatic actions might explain the performance of these countries. In Brazil, the 2002 National Policy on Health Care for Indigenous Peoples has promoted access to integrated health care through actions such as the organisation of health services in Special Health Districts where indigenous populations live, and intercultural training for health workers.^42^ In Honduras, the creation of a State Secretariat for Indigenous Peoples and Afro-Hondurans and the Strategic Plan for Integrated Development with Identity of Indigenous Peoples have led to important advances in indigenous health.^10^ In Mexico, conditional cash-transfer programmes allied to a policy on the Health Strategy of the Indigenous Peoples have had a positive effect on education and health indicators.^10^, ^43^, ^44^ In Suriname, government policies in place since the 1960s provide free access to primary and secondary health care for rural populations.^45^ Most of the indigenous population live in rural areas where free health services are available through the Medical Mission, a publicly subsidised non-governmental organisation.

Differences in coverage between people of African descent and the reference group were not as marked or consistent as those for indigenous women and children. Of the 33 analyses, when considering point estimates alone, only one coverage ratio was below 0·7 (contraception in Suriname), five were between 0·7 and 0·9, 26 were between 0·9 and 1·1, and one was greater than 1·1 (DPT3 vaccine, also in Suriname). Adjustment for wealth, education, and residence had small and inconsistent results, sometimes increasing or decreasing the estimates. Although, in most countries, people of African descent were considerably poorer than the reference category, levels of coverage similar to those for reference groups suggest that these countries have succeeded in reaching people of African descent with such basic interventions. It is worth noting that people of African descent—unlike the indigenous populations—are predominantly urban in all countries under study except in Guyana, and urban residence facilitates geographical access to health services. The concentration of people of African descent in urban areas is a well known characteristic of the region^46^ that is confirmed by our analyses (appendix).

The four interventions under study are not new. They have been available for decades in the region, which has resulted in high coverage in the reference groups in most countries (appendix). Therefore, a substantial amount of time has elapsed to allow these interventions to trickle down to excluded groups, after high coverage was achieved among the wealthier populations.^47^ This might not be the case for interventions that have been implemented more recently, such as mammography or cervical cancer screening, for which ethnic gaps might be wider than for the four interventions studied here.

In summary, our analyses of data from 16 countries revealed important gaps in RMNCH coverage between indigenous and non-indigenous populations. These inequities are partly explained by socioeconomic position and place of residence, but even after allowing for these factors, the gaps persist. Inequalities between people of African descent and the reference groups are less marked than for indigenous women and children. Continued monitoring of ethnic inequalities is essential to assess the impact of inclusive policies in the region and to guide future policy and implementation initiatives.

For more on **Countdown to 2030** see http://countdown2030.org
