# Supplementary material for: Ethnic group inequalities in coverage with reproductive, maternal and child health interventions: cross-sectional analyses of national surveys in 16 Latin American and Caribbean countries
Source: Lancet Glob Health. 2018 Jul 14;6(8):e902–13. doi: 10.1016/S2214-109X(18)30300-0 (PMC6057134; doi:10.1016/S2214-109X(18)30300-0)
Supplement: Supplementary appendix [file mmc1.pdf]

THE LANCET
Global Health

Supplementary appendix

This appendix formed part of the original submission and has been peer reviewed. We post it as supplied by the authors.

Supplement to: Mesenburg MA, Restrepo-Mendez MC, Amigo H, et al. Ethnic group inequalities in coverage with reproductive, maternal and child health interventions: cross-sectional analyses of national surveys in 16 Latin American and Caribbean countries. *Lancet Glob Health* 2018; **6:** e902–13.

THE LANCET Global Health

Supplementary appendix

This appendix formed part of the original submission and has been peer reviewed. We post it as supplied by the authors.

Supplement to: Mesenburg, et al. Ethnic group inequalities in coverage with reproductive, maternal and child health interventions: cross-sectional analyses of national surveys in 16 Latin America and the Caribbean countries. Lancet Glob Health 2017.

SUPPLEMENTARY APPENDIX

Index to the supplementary appendix

Tables

Figure S1 - Countries included in the analyses. 4

References to the final reports of the surveys included in the analyses. 5

Table S1 - Percentage of missing data for countries included in the analyses. 7

Panel - Ethnic groups in Suriname and Guyana 8

Table S2 - Intervention coverage and sample size by ethnic group for countries included in the analyses. 10

Figure S2 - Distribution of reference, afrodescendants and indigenous groups according to women's education, by country. 12

Figure S3 - Distribution of reference, afrodescendants and indigenous groups according to area of residence, by country. 13

Table S3 - Distribution of women aged 15-49 years in each ethnic group according to wealth, schooling and urban-rural residence. 14

Table S4 - Crude and adjusted coverage ratios (95%CI) for modern contraceptive use in indigenous women, compared to the reference category, by country. 16

Table S5 - Crude and adjusted coverage ratios (95%CI) for antenatal care (four or more visits) in indigenous women, compared to the reference category, by country. 17

Table S6 - Crude and adjusted coverage ratios (95%CI) for skilled birth attendant in indigenous women, compared to the reference category, by country. 18

Table S7 - Crude and adjusted coverage ratios (95%CI) for DPT immunization in indigenous children, compared to the reference category, by country. 19

Table S8 - Crude and adjusted coverage ratios (95%CI) for modern contraceptive use in afrodescendant women, compared to the reference. 20

Table S9 - Crude and adjusted coverage ratios (95%CI) for antenatal care (four or more visits) in afrodescendant women, compared to the reference. 21

Table S10 - Crude and adjusted coverage ratios (95%CI) for skilled birth attendant in afrodescendant women, compared to the reference category, by country. 22

Table S11 - Crude and adjusted coverage ratios (95%CI) for DPT immunization in afrodescendant children, compared to the reference category, by country. 23

Table S12 - Meta-analysis and meta-regression results for RMNCH interventions according to ethnic group in Latin America and the Caribbean. Co-variates include GDP per capita, urbanization and ethnic group composition of the countries under study. 24

Table S13 - Indigenous groups and proportion of population in recent national censuses in the countries included in the analyses. 25

Table S14 - Legal standpoint of indigenous people in the countries included in the analyses. .. 32

Table S15 – P values for comparisons presented in the Figure 2 and Figure 3 of the manuscript. 34

Table S16 – Intervention coverage by ethnic group (white, brown, black and indigenous), Brazil, 2006. 35

Figure S1 - Countries included in the analyses.

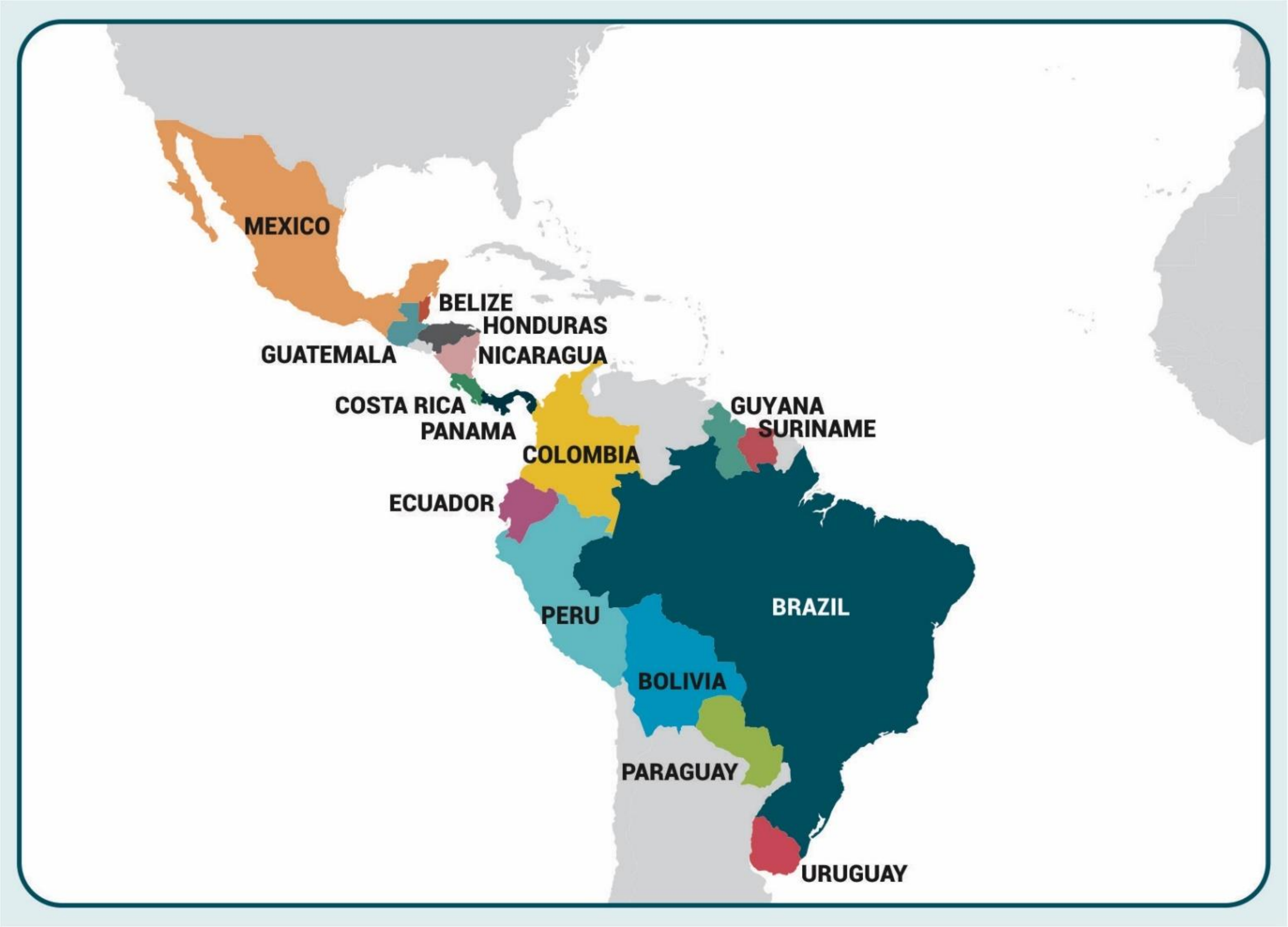

References to the final reports of the surveys included in the analyses.

DHS

1. Coa R, Ochoa LH. Bolivia: Encuesta Nacional de Demografía y Salud: ENDSA 2008. Calverton, Maryland: Ministerio de Salud y Deportes and Macro International.; 2009. Available from: https://dhsprogram.com/pubs/pdf/FR228/FR228[08Feb2010].pdf.

2. Instituto Nacional de Estadística e Informática. Perú Encuesta Demográfica y de Salud Familiar - ENDES 2012. Lima, Perú: INEI/Perú; 2013. Available from: http://dhsprogram.com/pubs/pdf/FR284/FR284.pdf.

3. Ministerio de Salud Pública y Asistencia Social, Instituto Nacional de Estadística, Secretaría de Planificación y Programación del la Presidencia, ICF International. Guatemala. Encuesta Nacional de Salud Materno Infantil 2014-2015: informe final. Rockville, Maryland: MSPAS, INE, Segeplán and ICF International; 2017. Available from: http://dhsprogram.com/pubs/pdf/FR318/FR318.pdf.

4. Ojeda G, Ordonez M, Ochoa LH. Colombia Encuesta Nacional de Demografía y Salud 2010. Bogotá, Colombia: Profamilia; 2011. Available from: https://dhsprogram.com/pubs/pdf/FR246/FR246.pdf.

5. Secretaría de Salud, Instituto Nacional de Estadística, ICF International. Honduras Encuesta Nacional de Salud y Demografía 2011-2012. Tegucigalpa, Honduras: SS, INE and ICF International; 2013. Available from: http://dhsprogram.com/pubs/pdf/FR274/FR274.pdf.

MODIFIED DHS

1. Brasil, Ministério da Sáude. PNDS 2006 Pesquisa Nacional de Demografia e Saúde da Criança e da Mulher. Brasília: MS; 2008. Available from: http://bvsms.saude.gov.br/bvs/publicacoes/pnds_crianca_mulher.pdf.

MICS

1. Bureau of Statistics, Ministry of Health, UNICEF (United Nations Children's Fund). Guyana: Multiple Indicator Cluster Survey 2014. Key Findings. Georgetown: Bureau of Statistics, Ministry of Health and UNICEF; 2015. Available from: https://goo.gl/ApDzzJ.

2. Contraloría General de la República. Panamá: Encuesta de Indicadores Múltiples por Conglomerados, 2013. Resultados principales. Panamá: Contraloría General 2014. Available from: https://goo.gl/vjakjR.

3. Ministry of Social Affairs and Housing, General Bureau of Statistics. Suriname: Multiple Indicator Cluster Survey 2010. Final Report. Paramaribo: MSAH; 2012. Available from: https://goo.gl/tGVAHt.

4. República de Costa Rica Ministerio de Salud, UNICEF (United Nations Children's Fund). Costa Rica: Encuesta de Indicadores Múltiples por Conglomerados. San José: Ministerio de Salud; 2013. Available from: https://goo.gl/qAfEU1.

5. Statistical Institute of Belize. Belize: Multiple Indicator Cluster Survey 2011. Final Report. New York: UNICEF; 2012. Available from: https://goo.gl/5LJ2qT.

6. UNICEF (United Nations Children's Fund). Mexico. Encuesta Nacional de Niños, Niñas y Mujeres - Encuesta de Indicadores Múltiples por Conglomerados 2015, Informe Final. Ciudad de México: Instituto Nacional de Salud Pública y UNICEF México; 2016. Available from: https://goo.gl/W5wHN3.

7. UNICEF (United Nations Children's Fund), MIDES (Ministerio de Desarrollo Social). Uruguay. Encuesta de Indicadores Múltiples por Conglomerados 2013: informe final. Montevideo: UNICEF, MIDES; 2015. Available from: https://goo.gl/wNbp4z.

RHS

1. Centro Paraguayo de Estudios de Población, Agencia del Gobierno de los Estados Unidos para el Desarrollo Internacional. Paraguay. Encuesta Nacional de Demografía y Salud Sexual y Reproductiva 2008 ENDSSR 2008: Informe Final. Asunción: CEPEP; 2009. Available from: http://www.cepep.org.py/archivos/ENDSSR%202008_1.pdf.

2. Ministerio de Salud Pública, Centro de Estudios de Población y Desarrollo Social, Instituto Nacional de Estadística y Censos. Ecuador: Encuesta Demográfica y de Salud Materna e Infantil - ENDEMAIN 2004. Quito: CEPAR; 2005. Available from: https://www.measureevaluation.org/resources/publications/tr-05-30-es.

3. Republica de Nicaragua, Instituto Nacional de Información de Desarrollo, Ministerio de Salud. Nicaragua. Encuesta Nicaragüense de Demografía y Salud ENDESA 2006/07: Informe Final. Managua: INIDE; 2008. Available from: http://www.inide.gob.ni/endesa/Endesa_2006/InformeFinal06_07.pdf.

Table S1 - Percentage of missing data for countries included in the analyses.

Country	year	CPMO	ANC4	SBA	DPTV
**Belize**	2011	8.1	11.4	0.0	3.6
**Bolivia**	2008	0.0	0.3	0.2	1.0
**Brazil**	2006	7.8	4.1	0.3	NA
**Colombia**	2010	0.0	1.2	0.0	1.3
**Costa Rica**	2011	4.7	1.3	0.0	0.2
**Ecuador**	2004	0.0	0.3	0.6	6.2
**Guatemala**	2014	0.0	0.1	<0.1	<0.1
**Guyana**	2014	8.5	7.2	0.6	2.3
**Honduras**	2011	0.0	0.1	0.0	0.7
**Mexico**	2015	4.4	0.1	<0.1	0.6
**Nicaragua**	2006	0.0	0.6	0.0	7.8
**Panama**	2013	6.8	0.6	<0.1	1.1
**Paraguay**	2008	3.1	0.2	2.1	0.0
**Peru**	2012	0.0	0.1	0.0	2.8
**Suriname**	2010	6.2	26.3	1.1	4.9
**Uruguay**	2012	NA	5.5	0.0	NA

CPMO: modern contraception; ANC4: Antenatal care (four or more visits); SBA: skilled birth attendant; DPTV: DPT vaccine.

Panel - Ethnic groups in Suriname and Guyana

In contrast to the predominantly Spanish and Portuguese colonization in the 14 countries included in the present analyses, Guyana and Suriname were colonized by the British and Dutch, respectively, and as a result show different ethnic mixes. In both countries, it was possible to identify indigenous and afrodescendants women and children, but the reference group representing the rest of the population differed from those in the rest of the region.

In Suriname, the MICS questionnaire included eight options, with the respective proportions of women aged 15-49 years in the sample: Indian (30.1%), Javanese (14.1%), mixed race (10.1%), indigenous/Amerindians (4.0%), creole (17.2%), maroon (24.5%). Women who reported being (East) Indian, Javanese or of mixed race constituted the reference category.

The afrodescendants in Suriname consist out of two separate groups: maroon and creole. Where creoles are descendants from plantation slaves, and maroons, descendants from runaway slaves who formed independent settlements in the hinterland far from the cities.

At present creoles adapted western styles and live in urban areas and maroons live like the indigenous in their traditional way.[1] In the 1990s, political disturbances caused many maroons to move from their natural living areas to periurban slums.[2-4] These displaced maroon groups are socio-economically disadvantaged. Maroon women have high fertility rates, and reportedly benefit from delivering children across the border in the French Guiana where health care is free and child support is available; this may explain their low use of contraceptives. The table below shows coverage levels and 95% confidence intervals for marron and creole women and children with the four interventions studied:

Intervention	Maroon	Creole
**Modern contraceptives**	27.3% (23.8 - 31.1)	42.7% (37.1 - 48.5)
**Antenatal care**	58.7% (54.1 - 63.1)	70.0% (58.5 - 77.9)
**Skilled attendant at birth**	88.1% (85.5 - 90.2)	97.1% (90.5 - 99.1)
**DPT3 vaccine**	61.9% (55.7 - 67.7)	52.0% (36.7 - 66.9)

Health services in the hinterland of Suriname are available through the medical mission, a government subsidized NGO established by missionaries which now serves tens of clinics. These provide primary health care staffed with health workers under the supervision of a physician through radio and monthly visits. This may explain the higher vaccine coverage among indigenous and maroon children than for the reference group.

In Guyana, four ethnic categories were recorded in the MICS questionnaire: East Indians (45.7%), mixed race (17.3%), Amerindians (6.8%) and African (30.2%). Guyana also has small numbers of Portuguese and Chinese descendants, who jointly account for about 3% of the population according to the most recent census. In the MICS database, these groups were included in the category of missing/ does not know/ other women, and were not included in the present analyses.[5]

References

1. Helman A. Cultureel mozaïek van Suriname. Zutphen: Walburg Pers; 1977.
2. Franszoon. The Suriname Maroons Crisis. 1988. [Available from: https://www.culturalsurvival.org/publications/cultural-survival-quarterly/suriname-maroon-crisis].
3. Price R. Maroons in Suriname and Guyana: how many and where. *New West Indian Guide* 2002; **76**(1/2): 81-8.
4. Price R. The Maroon population explosion: Suriname and Guyana. *New West Indian Guide* 2013; **76**(3/4): 323-27.
5. Government of Guyana Agency. Compendium 2: population composition. 2017. [Available from: http://www.statisticsguyana.gov.gy/census.html#comp].

Table S2 - Intervention coverage and sample size by ethnic group for countries included in the analyses.

Country/year	Ethnic group	Coverage							
		CPMO	Number	ANC4	Number	SBA	Number	DPTV	Children
**Belize**	Reference	54.3	1,423	87.2	416	96.5	416	74.6	234
**2011**	Indigenous	36.0	358	68.5	126	92.0	126	73.1	75
	Afrodescendants	52.6	573	80.0	152	97.6	152	77.9	85
**Brazil**	Reference	76.6	3,909	92.6	937	98.3	990	0.0	0
**2006**	Indigenous	72.5	205	82.9	62	91.4	70	0.0	0
	Afrodescendants	75.1	5,532	89.3	1,578	96.6	1,728	0.0	0
**Bolivia**	Reference	46.3	4,421	83.7	1,940	90.8	2,159	88.5	688
**2008**	Indigenous	26.5	5,757	64.7	2,684	62.9	3,062	84.5	993
	Afrodescendants	0.0	0	0.0	0	0.0	0	0.0	0
**Colombia**	Reference	73.9	21,525	89.0	7,031	97.2	7,557	91.5	2,450
**2010**	Indigenous	61.4	2,952	72.4	1,444	73.2	1,615	87.6	546
	Afrodescendants	68.5	2,855	84.7	1,214	91.2	1,329	85.3	438
**Costa Rica**	Reference	75.6	2,402	91.7	672	99.1	672	94.9	364
**2011**	Indigenous	66.7	155	56.0	50	90.2	50	88.2	27
	Afrodescendants	66.5	116	96.4	37	100.0	37	91.2	20
**Ecuador**	Reference	61.3	4,700	68.6	1,935	74.8	2,231	77.2	727
**2004**	Indigenous	24.3	526	27.2	316	25.3	403	65.9	123
	Afro-descendants	57.4	187	65.1	104	68.5	129	73.8	41
**Guatemala**	Reference	57.7	9,062	87.3	3,849	84.1	4,199	87.6	1,329
**2014**	Indigenous	35.9	5,903	84.2	2,877	49.7	3,251	81.5	1,072
	Afrodescendants	0.0	0	0.0	0	0.0	0	0.0	0
**Guyana**	Reference	34.4	2,164	88.9	655	96.7	655	92.7	352
**2014**	Indigenous	31.2	571	77.1	272	61.7	272	88.2	144
	Afrodescendants	29.1	1,009	87.2	326	99.0	326	91.8	174
**Honduras**	Reference	64.0	10,485	88.2	4,845	84.9	5,255	95.8	1,786
**2011**	Indigenous	61.2	1,904	87.0	990	78.4	1,085	95.3	365
	Afrodescendants	68.4	348	94.2	163	89.6	184	97.6	59
**Mexico**	Reference	64.7	6,624	95.3	2,543	98.6	2,543	72.0	152
**2015**	Indigenous	57.3	768	81.6	297	87.9	297	77.1	1,279
	Afro-descendants	0.0	0	0.0	0	0.0	0	0.0	0
**Nicaragua**	Reference	69.3	8,481	80.9	3,509	77.9	3,792	96.4	1,281
**2006**	Indigenous	57.1	367	57.2	221	52.6	248	80.3	76
	Afrodescendants	0.0	0	0.0	0	0.0	0	0.0	0
**Panama**	Reference	64.5	3,170	92.7	1,017	98.3	1,017	86.0	577
**2013**	Indigenous	29.2	2,072	69.2	950	66.5	950	72.6	544
	Afrodescendants	64.2	873	94.5	311	99.1	311	73.9	188
**Paraguay**	Reference	71.2	2,269	96.8	889	92.6	958	77.8	311
**2008**	Indigenous	65.7	1,480	86.0	681	76.8	762	66.9	226
	Afrodescendants	0.0	0	0.0	0	0.0	0	0.0	0
**Peru**	Reference	53.2	12,488	94.7	4,656	90.5	4,964	83.9	1,634

**2012**	Indigenous	36.5	1,739	89.3	674	68.0	741	81.9	257
	Afrodescendants	0.0	0	0.0	0	0.0	0	0.0	0
**Suriname**	Reference	54.4	1,925	74.7	371	96.2	371	51.4	209
**2010**	Indigenous	43.8	259	73.6	78	90.9	78	76.8	53
	Afrodescendants	34.0	1,216	61.1	800	90.2	800	59.9	434
**Uruguay**	Reference	0.0	0	88.1	328	99.6	328	0.0	0
**2012**	Indigenous	0.0	0	12.1	17	100.0	17	0.0	0
	Afrodescendants	0.0	0	78.0	43	95.6	43	0.0	0

CPMO: modern contraception; ANC4: Antenatal care (four or more visits); SBA: skilled birth attendant; DPTV: DPT vaccine.

Figure S2 - Distribution of reference, afrodescendants and indigenous groups according to women's education, by country.

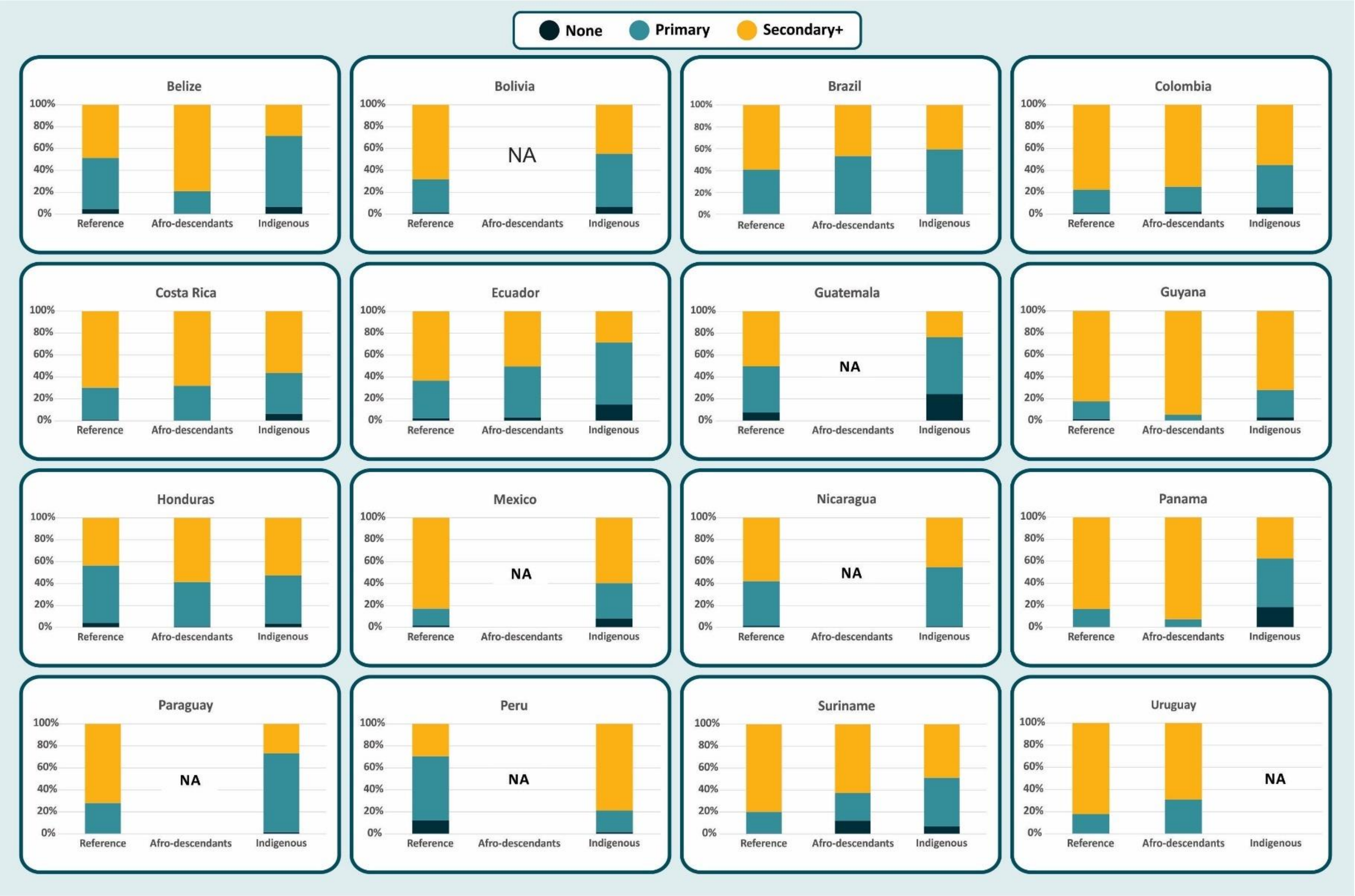

Figure S3 - Distribution of reference, afrodescendants and indigenous groups according to area of residence, by country.

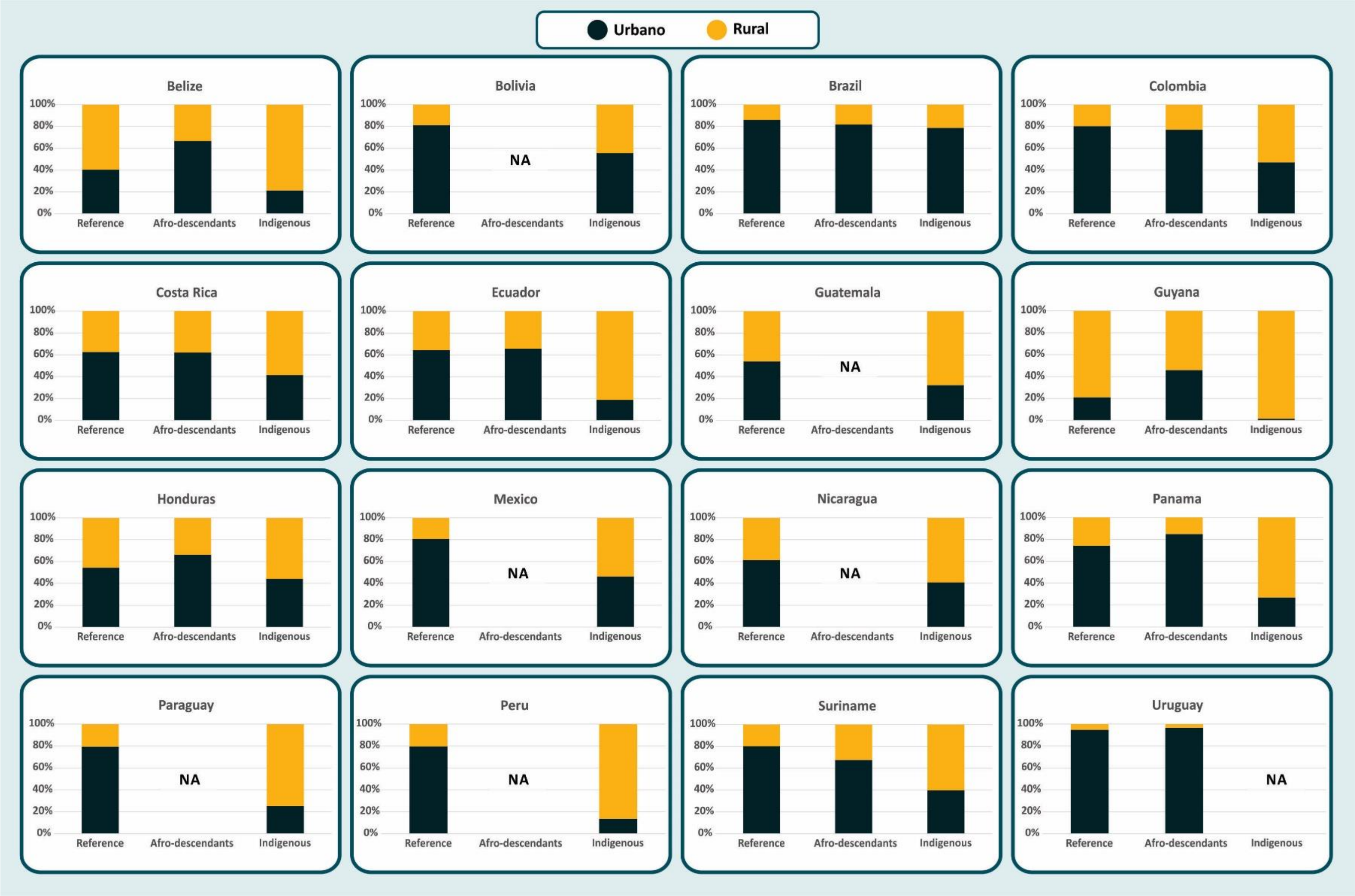

Table S3 - Distribution of women aged 15-49 years in each ethnic group according to wealth, schooling and urban-rural residence.

Country/year	Ethnic group	Total%	Wealth quintiles						Education				Residence		
			Poorest	2nd	3rd	4th	Richest	P level	None	Primary	Secondary+	P level	Urban	Rural	P level
**Belize**	Reference	59.1	14.8	22.1	21.2	20.5	21.4	<0.001	4.9	46.5	48.6	<0.001	40.4	59.6	<0.001
**2011**	Indigenous	10.1	52.4	20.8	14.1	9.9	2.8		6.7	64.8	28.5		21.4	78.6	
	Afrodescendants	30.8	6.0	15.7	24.5	25.0	28.8		0.5	20.5	79.0		66.8	33.2	
**Brazil**	Reference	41.6	13.0	15.6	18.2	22.9	30.3	<0.001	0.3	40.4	59.3	<0.001	86.0	14.0	<0.001
**2006**	Indigenous	2.0	26.8	20.6	17.8	19.7	15.1		0.6	58.8	40.6		78.7	21.3	
	Afrodescendants	56.4	24.6	22.8	21.1	17.8	13.7		0.8	52.6	46.8		81.7	18.3	
**Bolivia**	Reference	40.4	6.3	12.1	19.0	26.5	36.1	<0.001	1.6	30.2	68.2	<0.001	81.2	18.8	<0.001
**2008**	Indigenous	59.6	21.7	20.7	21.7	19.9	16.0		6.6	48.6	44.8		55.6	44.4	
	Afrodescendants	NA	NA	NA	NA	NA	NA		NA	NA	NA		NA	NA	
**Colombia**	Reference	85.7	13.9	19.1	22.1	22.8	22.1	<0.001	1.3	21	77.7	<0.001	80.2	19.8	<0.001
**2010**	Indigenous	3.9	50.9	19.5	15.2	8.7	5.7		6.4	38.5	55.1		47.1	52.9	
	Afrodescendants	10.4	26.8	23.3	19.5	17.7	12.7		2.3	22.9	74.8		76.9	23.1	
**Costa Rica**	Reference	92.9	16.2	19.2	20.6	21.6	22.4	<0.001	0.9	29.4	69.7	<0.001	62.5	37.5	<0.001
**2011**	Indigenous	4.7	39.4	16.6	11.7	13.6	18.7		6.4	37.1	56.5		41.5	58.5	
	Afrodescendants	2.4	15.6	27.0	24.3	16.1	17.0		0.3	31.6	68.1		62.1	37.9	
**Ecuador**	Reference	89.4	13.3	18.4	20.8	21.5	26.0	<0.001	2.4	34.4	63.2	<0.001	64.3	35.7	<0.001
**2004**	Indigenous	7.5	51.7	24.4	12.8	7.8	3.3		15.0	56.5	28.5		18.8	81.7	
	Afrodescendants	3.1	17.5	27.0	22.2	17.0	16.3		3.2	46.5	50.3		65.7	34.3	
**Guatemala**	Reference	60.1	8.3	13.7	19.4	26.7	31.9	<0.001	7.6	42.2	50.2	<0.001	53.9	46.1	<0.001
**2014**	Indigenous	39.9	31.0	26.3	20.7	14.6	7.4		24.3	52.0	23.7		32.3	67.7	
	Afrodescendants	NA	NA	NA	NA	NA	NA		NA	NA	NA		NA	NA	
**Guyana**	Reference	63.1	11.9	19.1	21.2	23.0	24.8	<0.001	1.5	16.1	82.4	<0.001	21.1	78.9	<0.001
**2014**	Indigenous	6.8	79.5	9.9	5.5	3.8	1.3		3.2	24.8	72.0		1.6	98.4	
	Afrodescendants	30.1	13.7	18.9	20.1	25.1	22.2		0.0	5.5	94.5		45.8	54.2	

**Honduras**	Reference	87.0	15.3	17.9	21.5	23.4	21.9	<0.001	4.2	52.3	43.5	<0.001	54.4	45.6	<0.001
**2011**	Indigenous	10.5	26.6	21.8	16.8	15.2	19.6		3.6	43.6	52.8		44.3	55.7	
	Afrodescendants	2.5	7.5	12.5	22.2	27.2	30.6		0.8	40.6	58.6		66.2	33.8	
**Mexico**	Reference	92.5	14.7	20.9	21.8	21.1	21.5	<0.001	1.5	15.6	82.9	<0.001	80.6	19.4	<0.001
**2015**	Indigenous	7.5	66.9	14.5	11.0	4.7	2.9		7.9	32.5	59.6		46.2	53.8	
	Afrodescendants	NA	NA	NA	NA	NA	NA		NA	NA	NA		NA	NA	
**Nicaragua**	Reference	95.6	17.4	19.0	20.1	22.1	21.4	<0.001	1.4	40.8	57.8	<0.001	61.2	38.8	<0.001
**2006**	Indigenous	4.4	45.4	18.6	19.3	12.9	3.8		1.0	53.9	45.1		40.9	59.1	
	Afrodescendants	NA	NA	NA	NA	NA	NA		NA	NA	NA		NA	NA	
**Panama**	Reference	71.8	10.7	19.9	22.5	22.1	24.8	<0.001	0.3	16.3	83.4	<0.001	74.2	25.8	<0.001
**2013**	Indigenous	10.9	76.7	16.0	5.8	1.2	0.3		18.5	44.1	37.4		26.8	73.2	
	Afrodescendants	17.3	6.2	16.1	20.0	31.8	25.9		0.2	7.2	92.6		84.8	15.2	
**Paraguay**	Reference	21.2	7.7	16.5	22.3	25.7	27.8	<0.001	0.1	28.0	71.9	<0.001	79.5	20.5	<0.001
**2008**	Indigenous	71.8	51.2	28.5	13.8	4.3	2.2		1.6	71.6	26.8		25.3	74.7	
	Afrodescendants	NA	NA	NA	NA	NA	NA		NA	NA	NA		NA	NA	
**Peru**	Reference	92.6	11.5	17.9	23.0	23.7	23.9	<0.001	12.2	58.4	29.4	<0.001	79.7	20.3	<0.001
**2012**	Indigenous	7.4	63	30.9	4.8	1.3	0.0		1.7	19.6	78.7		13.6	86.4	
	Afrodescendants	NA	NA	NA	NA	NA	NA		NA	NA	NA		NA	NA	
**Suriname**	Reference	54.3	5.2	19.0	22.1	26.2	27.5	<0.001	0.5	19.7	79.8	<0.001	80.0	20.0	<0.001
**2010**	Indigenous	4.0	47.8	18.3	17.9	8.1	7.9		6.9	44.4	48.7		39.7	60.3	
	Afrodescendants	41.7	32.0	20.6	17.7	15.5	14.2		12.1	25.3	62.6		67.5	32.5	
**Uruguay**	Reference	88.0	16.1	17.0	19.7	26.0	21.2	<0.001	0.0	17.8	82.2	0.228	94.7	5.3	0.098
**2012**	Indigenous	4.9	11.3	70.7	6.2	6.3	5.5		0.0	37.7	62.3		98.7	1.3	
	Afrodescendants	7.1	42.8	30.7	13.9	9.5	3.1		0.0	30.9	69.1		96.7	3.3	

NA: not available.

Table S4 - Crude and adjusted coverage ratios (95%CI) for modern contraceptive use in indigenous women, compared to the reference category, by country.

Country/year	Crude CR	Adjusted for			
		Wealth	Area	Education	All stratfiers
**Belize**	0.66	0.72	0.68	0.65	0.69
**2011**	0.56 - 0.78	0.60 - 0.86	0.57 - 0.80	0.55 - 0.77	0.57 - 0.82
**Bolivia**	0.57	0.64	0.62	0.62	0.65
**2008**	0.54 - 0.61	0.60 - 0.68	0.58 - 0.66	0.58 - 0.66	0.61 - 0.70
**Brazil**	0.95	0.96	0.95	0.94	0.95
**2006**	0.83 - 1.09	0.84 - 1.10	0.83 - 1.08	0.81 - 1.08	0.82 - 1.10
**Colombia**	0.83	0.85	0.83	0.84	0.85
**2010**	0.79 - 0.87	0.81 - 0.89	0.79 - 0.87	0.80 - 0.88	0.81 - 0.90
**Costa Rica**	0.88	0.91	0.89	0.89	0.92
**2011**	0.72 - 1.09	0.75 - 1.11	0.72 - 1.10	0.73 - 1.10	0.76 - 1.12
**Ecuador**	0.40	0.44	0.42	0.43	0.46
**2004**	0.33 - 0.48	0.37 - 0.53	0.35 - 0.51	0.35 - 0.51	0.38 - 0.55
**Guatemala**	0.62	0.71	0.65	0.66	0.72
**2014**	0.60 - 0.65	0.68 - 0.75	0.62 - 0.68	0.63 - 0.69	0.68 - 0.75
**Guyana**	0.91	0.97	0.90	0.92	0.95
**2014**	0.77 - 1.07	0.78 - 1.19	0.76 - 1.07	0.77 - 1.09	0.77 - 1.18
**Honduras**	0.96	0.98	0.97	0.95	0.98
**2011**	0.91 - 1.00	0.93 - 1.03	0.92 - 1.01	0.91 - 1.00	0.93 - 1.03
**Mexico**	0.88	0.94	0.90	0.89	0.94
**2015**	0.79 - 0.99	0.84 - 1.05	0.80 - 1.00	0.79 - 0.99	0.84 - 1.05
**Nicaragua**	0.82	0.86	0.83	0.82	0.84
**2006**	0.74 - 0.91	0.77 - 0.95	0.75 - 0.93	0.73 - 0.92	0.75 - 0.95
**Panama**	0.45	0.47	0.44	0.49	0.49
**2013**	0.40 - 0.52	0.40 - 0.55	0.38 - 0.51	0.42 - 0.57	0.41 - 0.58
**Paraguay**	0.92	0.92	0.91	0.95	0.93
**2008**	0.88 - 0.97	0.86 - 0.98	0.86 - 0.97	0.89 - 1.01	0.87 - 1.00
**Peru**	0.69	0.76	0.75	0.74	0.79
**2012**	0.64 - 0.74	0.71 - 0.83	0.69 - 0.81	0.69 - 0.80	0.73 - 0.85
**Suriname**	0.81	0.87	0.80	0.86	0.90
**2010**	0.68 - 0.96	0.71 - 1.06	0.67 - 0.95	0.72 - 1.03	0.73 - 1.10

Table S5 - Crude and adjusted coverage ratios (95%CI) for antenatal care (four or more visits) in indigenous women, compared to the reference category, by country.

Country/year	Crude CR	Adjusted for			
		Wealth	Area	Education	All stratfiers
**Belize**	0.79	0.84	0.79	0.78	0.80
**2011**	0.69 - 0.89	0.73 - 0.97	0.69 - 0.90	0.68 - 0.89	0.70 - 0.92
**Bolivia**	0.77	0.87	0.83	0.83	0.88
**2008**	0.74 - 0.80	0.83 - 0.90	0.80 - 0.87	0.78 - 0.86	0.84 - 0.91
**Brazil**	0.89	0.91	0.90	0.89	0.91
**2006**	0.75 - 1.07	0.77 - 1.08	0.75 - 1.07	0.76 - 1.05	0.76 - 1.07
**Colombia**	0.81	0.87	0.84	0.86	0.89
**2010**	0.77 - 0.86	0.82 - 0.92	0.80 - 0.89	0.82 - 0.91	0.84 - 0.94
**Costa Rica**	0.61	0.62	0.61	0.61	0.61
**2011**	0.39 - 0.96	0.40 - 0.95	0.37 - 0.97	0.38 - 0.97	0.40 - 0.94
**Ecuador**	0.40	0.51	0.46	0.46	0.52
**2004**	0.32 - 0.50	0.41 - 0.64	0.37 - 0.58	0.37 - 0.57	0.42 - 0.66
**Guatemala**	0.96	1.00	0.97	0.99	1.00
**2014**	0.94 - 0.99	0.97 - 1.02	0.95 - 0.99	0.96 - 1.01	0.98 - 1.03
**Guyana**	0.87	0.89	0.86	0.89	0.89
**2014**	0.80 - 0.94	0.81 - 0.98	0.79 - 0.94	0.82 - 0.96	0.81 - 0.98
**Honduras**	0.99	1.00	0.99	0.98	1.00
**2011**	0.96 - 1.02	0.98 - 1.03	0.96 - 1.02	0.95 - 1.01	0.97 - 1.02
**Mexico**	0.86	0.88	0.86	0.88	0.89
**2015**	0.79 - 0.92	0.82 - 0.95	0.79 - 0.93	0.82 - 0.94	0.83 - 0.96
**Nicaragua**	0.71	0.78	0.74	0.76	0.81
**2006**	0.62 - 0.81	0.69 - 0.89	0.64 - 0.84	0.66 - 0.88	0.71 - 0.92
**Panama**	0.75	0.81	0.78	0.86	0.89
**2013**	0.70 - 0.80	0.74 - 0.88	0.72 - 0.84	0.80 - 0.92	0.82 - 0.96
**Paraguay**	0.89	0.95	0.91	0.93	0.97
**2008**	0.86 - 0.92	0.92 - 0.99	0.87 - 0.95	0.90 - 0.97	0.93 - 1.01
**Peru**	0.94	0.99	0.97	0.98	1.00
**2012**	0.92 - 0.97	0.96 - 1.03	0.94 - 1.00	0.95 - 1.01	0.97 - 1.03
**Suriname**	0.99	0.99	0.94	1.01	0.98
**2010**	0.82 - 1.18	0.81 - 1.21	0.78 - 1.14	0.83 - 1.22	0.80 - 1.20

Table S6 - Crude and adjusted coverage ratios (95%CI) for skilled birth attendant in indigenous women, compared to the reference category, by country.

Country/year	Crude CR	Adjusted for			
		Wealth	Area	Education	All stratfiers
**Belize**	0.95	0.99	0.95	0.96	0.98
**2011**	0.91 - 1.00	0.94 - 1.04	0.91 - 1.00	0.91 - 1.01	0.93 - 1.04
**Bolivia**	0.69	0.82	0.79	0.76	0.84
**2008**	0.67 - 0.72	0.80 - 0.85	0.77 - 0.82	0.74 - 0.79	0.81 - 0.86
**Brazil**	0.93	0.93	0.93	0.94	0.94
**2006**	0.84 - 1.03	0.85 - 1.03	0.84 - 1.03	0.85 - 1.03	0.85 - 1.03
**Colombia**	0.75	0.79	0.78	0.78	0.81
**2010**	0.72 - 0.79	0.76 - 0.83	0.74 - 0.82	0.75 - 0.82	0.77 - 0.85
**Costa Rica**	0.91	0.92	0.91	0.91	0.91
**2011**	0.81 - 1.02	0.82 - 1.03	0.82 - 1.02	0.81 - 1.02	0.82 - 1.02
**Ecuador**	0.34	0.43	0.41	0.40	0.47
**2004**	0.27 - 0.42	0.35 - 0.53	0.33 - 0.50	0.33 - 0.50	0.38 - 0.57
**Guatemala**	0.59	0.72	0.62	0.66	0.74
**2014**	0.57 - 0.62	0.69 - 0.75	0.60 - 0.65	0.64 - 0.69	0.71 - 0.77
**Guyana**	0.63	0.67	0.64	0.64	0.68
**2014**	0.56 - 0.71	0.60 - 0.75	0.57 - 0.72	0.57 - 0.72	0.61 - 0.76
**Honduras**	0.92	0.98	0.94	0.90	0.96
**2011**	0.89 - 0.96	0.95 - 1.01	0.91 - 0.97	0.88 - 0.93	0.93 - 1.00
**Mexico**	0.89	0.92	0.90	0.90	0.92
**2015**	0.85 - 0.94	0.87 - 0.96	0.86 - 0.95	0.86 - 0.95	0.88 - 0.96
**Nicaragua**	0.68	0.84	0.74	0.75	0.86
**2006**	0.59 - 0.78	0.74 - 0.96	0.66 - 0.85	0.65 - 0.85	0.76 - 0.97
**Panama**	0.68	0.76	0.72	0.79	0.83
**2013**	0.63 - 0.72	0.71 - 0.81	0.68 - 0.77	0.74 - 0.84	0.78 - 0.88
**Paraguay**	0.83	0.88	0.85	0.87	0.90
**2008**	0.79 - 0.88	0.83 - 0.93	0.80 - 0.90	0.82 - 0.93	0.84 - 0.95
**Peru**	0.75	0.92	0.88	0.86	0.96
**2012**	0.71 - 0.79	0.87 - 0.99	0.83 - 0.94	0.81 - 0.92	0.90 - 1.02
**Suriname**	0.95	0.98	0.95	0.95	0.98
**2010**	0.88 - 1.01	0.92 - 1.05	0.90 - 1.02	0.89 - 1.01	0.92 - 1.04

Table S7 - Crude and adjusted coverage ratios (95%CI) for DPT immunization in indigenous children, compared to the reference category, by country.

Country/year	Crude CR	Adjusted for			
		Wealth	Area	Education	All stratfiers
**Belize**	0.98	0.94	0.97	0.98	0.94
**2011**	0.83 - 1.16	0.79 - 1.13	0.81 - 1.14	0.83 - 1.15	0.79 - 1.13
**Bolivia**	0.95	0.95	0.94	0.97	0.95
**2008**	0.91 - 1.00	0.90 - 1.00	0.89 - 0.99	0.92 - 1.02	0.90 - 1.00
**Brazil**	NA	NA	NA	NA	NA
**2006**	NA	NA	NA	NA	NA
**Colombia**	0.96	0.98	0.97	0.97	0.99
**2010**	0.91 - 1.01	0.93 - 1.04	0.92 - 1.02	0.92 - 1.03	0.94 - 1.05
**Costa Rica**	0.93	0.97	0.93	0.92	0.95
**2011**	0.78 - 1.11	0.77 - 1.22	0.78 - 1.10	0.78 - 1.08	0.77 - 1.18
**Ecuador**	0.85	0.92	0.89	0.91	0.93
**2004**	0.72 - 1.01	0.78 - 1.08	0.76 - 1.06	0.78 - 1.07	0.79 - 1.09
**Guatemala**	0.93	0.96	0.93	0.95	0.97
**2014**	0.89 - 0.97	0.92 - 1.00	0.90 - 0.97	0.91 - 0.99	0.92 - 1.01
**Guyana**	0.95	0.96	0.96	0.96	0.96
**2014**	0.88 - 1.03	0.86 - 1.07	0.88 - 1.04	0.88 - 1.05	0.87 - 1.06
**Honduras**	0.99	0.99	0.99	0.99	0.99
**2011**	0.96 - 1.03	0.96 - 1.02	0.96 - 1.02	0.96 - 1.02	0.96 - 1.02
**Mexico**	1.07	1.06	1.02	1.08	1.05
**2015**	0.95 - 1.21	0.93 - 1.21	0.91 - 1.15	0.96 - 1.22	0.93 - 1.19
**Nicaragua**	0.83	0.87	0.85	0.84	0.86
**2006**	0.72 - 0.97	0.75 - 1.01	0.73 - 0.99	0.70 - 1.01	0.72 - 1.02
**Panama**	0.84	0.85	0.80	0.82	0.84
**2013**	0.75 - 0.95	0.73 - 0.99	0.71 - 0.90	0.72 - 0.94	0.72 - 0.98
**Paraguay**	0.86	0.95	0.83	0.94	0.93
**2008**	0.76 - 0.98	0.81 - 1.12	0.72 - 0.95	0.82 - 1.08	0.79 - 1.10
**Peru**	0.98	0.98	0.98	0.99	0.99
**2012**	0.91 - 1.04	0.91 - 1.05	0.91 - 1.06	0.92 - 1.06	0.91 - 1.06
**Suriname**	1.49	1.47	1.40	1.37	1.35
**2010**	1.19 - 1.86	1.13 - 1.91	1.11 - 1.77	1.06 - 1.79	1.03 - 1.78

NA: not available.

Table S8 - Crude and adjusted coverage ratios (95%CI) for modern contraceptive use in afrodescendant women, compared to the reference.

Country/year	Crude CR	Adjusted for			
		Wealth	Area	Education	All stratfiers
**Belize**	0.97	0.95	0.96	0.94	0.94
**2011**	0.88 - 1.07	0.86 - 1.04	0.87 - 1.06	0.85 - 1.04	0.85 - 1.04
**Brazil**	0.98	0.98	0.98	0.98	0.99
**2006**	0.94 - 1.02	0.94 - 1.03	0.94 - 1.02	0.94 - 1.02	0.94 - 1.03
**Colombia**	0.93	0.93	0.93	0.93	0.94
**2010**	0.90 - 0.96	0.90 - 0.96	0.90 - 0.96	0.90 - 0.96	0.91 - 0.97
**Costa Rica**	0.88	0.88	0.88	0.88	0.88
**2011**	0.67 - 1.15	0.68 - 1.15	0.67 - 1.15	0.67 - 1.15	0.68 - 1.15
**Ecuador**	0.94	0.96	0.93	0.94	0.95
**2004**	0.81 - 1.09	0.83 - 1.12	0.80 - 1.09	0.81 - 0.10	0.82 - 1.11
**Guyana**	0.85	0.84	0.85	0.84	0.84
**2014**	0.73 - 0.98	0.73 - 0.98	0.73 - 0.99	0.72 - 0.97	0.72 - 0.98
**Honduras**	1.07	1.04	1.05	1.06	1.04
**2011**	0.98 - 1.17	0.95 - 1.14	0.96 - 1.15	0.96 - 1.16	0.95 - 1.14
**Panama**	1.00	0.99	1.01	0.99	1.00
**2013**	0.90 - 1.10	0.89 - 1.10	0.91 - 1.12	0.89 - 1.10	0.90 - 1.11
**Suriname**	0.62	0.68	0.63	0.68	0.70
**2010**	0.56 - 0.69	0.60 - 0.76	0.57 - 0.70	0.61 - 0.76	0.63 - 0.79
**Uruguay**	NA	NA	NA	NA	NA
**2012**	NA	NA	NA	NA	NA

NA: not available.

Table S9 - Crude and adjusted coverage ratios (95%CI) for antenatal care (four or more visits) in afrodescendant women, compared to the reference.

Country/year	Crude CR	Adjusted for			
		Wealth	Area	Education	All stratfiers
**Belize**	0.92	0.89	0.90	0.86	0.85
**2011**	0.84 - 1.01	0.81 - 0.98	0.81 - 0.99	0.78 - 0.95	0.77 - 0.95
**Brazil**	0.96	0.97	0.98	0.97	0.97
**2006**	0.92 - 1.01	0.92 - 1.02	0.92 - 1.02	0.92 - 1.02	0.92 - 1.02
**Colombia**	0.95	0.98	0.96	0.96	0.98
**2010**	0.92 - 0.98	0.95 - 1.01	0.93 - 0.99	0.94 - 0.99	0.95 - 1.01
**Costa Rica**	1.05	1.02	1.05	1.05	1.02
**2011**	0.97 - 1.14	0.95 - 1.10	0.96- 1.14	0.97 - 1.14	0.95 - 1.10
**Ecuador**	0.95	1.00	0.93	1.00	1.01
**2004**	0.79 - 1.14	0.84 - 1.18	0.78 - 1.12	0.84 - 1.19	0.86 - 1.19
**Guyana**	0.98	0.98	0.98	0.97	0.98
**2014**	0.92 - 1.04	0.92 - 1.04	0.92 - 1.05	0.92 - 1.03	0.92 - 1.04
**Honduras**	1.07	1.05	1.06	1.06	1.04
**2011**	1.03 - 1.11	1.00 - 1.09	1.02 - 1.10	1.01 - 1.10	1.00 - 1.09
**Panama**	1.02	1.01	1.01	1.01	1.01
**2013**	0.96 - 1.08	0.95 - 1.07	0.96 - 1.08	0.95 - 1.07	0.95 - 1.07
**Suriname**	0.82	0.85	0.82	0.85	0.86
**2010**	0.74 - 0.90	0.76 - 0.96	0.74 - 0.91	0.76 - 0.95	0.77 - 0.97
**Uruguay**	0.89	0.94	0.89	0.90	0.95
**2012**	0.63 - 1.25	0.69 - 1.28	0.63 - 1.25	0.61 - 1.34	0.68 - 1.32

Table S10 - Crude and adjusted coverage ratios (95%CI) for skilled birth attendant in afrodescendant women, compared to the reference category, by country.

**Country/year**	Crude CR	Adjusted for			
		**Wealth**	**Area**	**Education**	**All stratfiers**
**Belize**	1.01	1.00	1.00	0.98	0.98
**2011**	0.98 - 1.04	0.97 - 1.03	0.97 - 1.04	0.95 - 1.02	0.94 - 1.02
**Brazil**	0.99	0.99	0.98	0.98	0.99
**2006**	0.97 - 1.01	0.97 - 1.00	0.97 - 1.00	0.97 - 1.00	0.97 - 1.00
**Colombia**	0.94	0.96	0.94	0.94	0.96
**2010**	0.92 - 0.95	0.94 - 0.98	0.93 - 0.96	0.93 - 0.96	0.94 - 0.97
**Costa Rica**	1.01	1.01	1.01	1.01	1.01
**2011**	1.00 - 1.02	1.00 - 1.02	1.00 - 1.01	1.00 - 1.01	1.00 - 1.02
**Ecuador**	0.92	0.94	0.89	0.97	0.94
**2004**	0.80 - 1.05	0.83 - 1.06	0.79 - 1.00	0.85 - 1.11	0.83 - 1.06
**Guyana**	1.02	1.02	1.02	1.02	1.02
**2014**	1.00 - 1.04	1.00 - 1.04	1.00 - 1.04	1.00 - 1.04	0.99 - 1.04
**Honduras**	1.06	0.99	1.02	1.03	0.98
**2011**	1.00 - 1.11	0.94 - 1.04	0.97 - 1.07	0.97 - 1.09	0.94 - 1.04
**Panama**	1.01	1.00	1.00	1.00	0.99
**2013**	1.00 - 1.02	0.99 - 1.01	0.99 - 1.01	0.99 - 1.01	0.99 - 1.00
**Suriname**	0.94	0.99	0.97	0.97	1.00
**2010**	0.91 - 0.97	0.96 - 1.03	0.94 - 1.00	0.94 - 1.00	0.97 - 1.04
**Uruguay**	0.96	0.96	0.96	0.95	0.95
**2012**	0.90 - 1.03	0.89 - 1.03	0.89 - 1.03	0.88 - 1.03	0.88 - 1.03

Table S11 - Crude and adjusted coverage ratios (95%CI) for DPT immunization in afrodescendant children, compared to the reference category, by country.

Country/year	Crude CR	Adjusted for			
		Wealth	Area	Education	All stratfiers
**Belize**	1.05	1.06	1.10	1.07	1.11
**2011**	0.91 - 1.20	0.92 - 1.23	0.94 - 1.28	0.92 - 1.24	0.95 - 1.30
**Brazil**	NA	NA	NA	NA	NA
**2006**	NA	NA	NA	NA	NA
**Colombia**	0.93	0.95	0.93	0.93	0.95
**2010**	0.89 - 0.98	0.91 - 0.99	0.89 - 0.98	0.89 - 0.98	0.91 - 1.00
**Costa Rica***					
**2011**					
**Ecuador**	0.96	0.97	0.93	0.98	1.00
**2004**	0.77 - 1.19	0.79 - 1.20	0.76 - 1.16	0.80 - 1.21	0.81 - 1.22
**Guyana**	0.99	0.99	0.99	0.98	0.98
**2014**	0.92 - 1.06	0.92 - 1.06	0.92 - 1.06	0.92 - 1.05	0.92 - 1.05
**Honduras**	1.02	1.02	1.02	1.02	1.02
**2011**	0.99 - 1.05	0.99 - 1.05	0.99 - 1.05	0.99 - 1.05	0.99 - 1.05
**Panama**	0.86	0.86	0.88	0.87	0.88
**2013**	0.72 - 1.03	0.72 - 1.03	0.74 - 1.05	0.72 - 1.04	0.74 - 1.05
**Suriname**	1.17	1.06	1.10	1.12	1.06
**2010**	0.98 - 1.39	0.86 - 1.31	0.91 - 1.32	0.92 - 1.37	0.86 - 1.31
**Uruguay**	NA	NA	NA	NA	NA
**2012**	NA	NA	NA	NA	NA

*Sample size < 25. NA: not available.

Table S12 - Meta-analysis and meta-regression results for RMNCH interventions according to ethnic group in Latin America and the Caribbean. Co-variates include GDP per capita, urbanization and ethnic group composition of the countries under study.

**Ethnic group**	**Indigenous**				**Afrodescendants**			
**Parameter/Indicator**	CPMO	ANC	SBA	DPT	CPMO	ANC	SBA	DPT
**Pooled effect (CI 95%)**	0.79 (0.72 - 0.87)	0.91 (0.87 - 0.95)	0.85 (0.80 - 0.90)	0.97 (0.95 - 1.00)	0.92 (0.87 - 0.99)	0.98 (0.94 - 1.01)	0.99 (0.98 - 1.00)	0.99 (0.95 - 1.03)
**I^2**	93.7	87.1	93.4	17.4	79.5	57.3	76.1	43.8
**Adjusted R^2**	*	46.1	*	*	*	*	26.8	*

(*) Model did not converge or R^2 value was negative, indicating that the explanatory variables did not account for the variability.

Table S13 - Indigenous groups and proportion of population in recent national censuses in the countries included in the analyses.

Country	Data source	Ethnic groups	Population	Percent of total population
**Belize**	2010 Census	Maya Ketchi	20616	6.4
		Maya Mopan	13022	4.0
		Maya Yucatec	2,869	0.9
		TOTAL	36507	11.2
**Bolivia**	2012 Census	Araona	910	<0.1
		Aymara	1,191,352	17.2
		Ayoreo	1,862	<0.1
		Baure	2,319	<0.1
		Canichana	617	<0.1
		Cavineño	2,005	<0.1
		Cayubaba	1,424	<0.1
		Chacobo	826	<0.1
		Chipaya	786	<0.1
		Chiquitano	87,885	1.3
		Esse Ejja	695	<0.1
		Guaraní	58,990	0.9
		Guarasugwe	42	<0.1
		Guarayo	13,621	0.2
		Itonama	10,275	0.1
		Joaquiniano	2,797	<0.1
		Kallawaya	7,389	0.1
		Leco	9,006	0.1
		Machinerí	38	<0.1
		Maropa	2,857	<0.1
		Mojeño	31,078	0.4
		Moré	155	<0.1
		Mosetén	1,989	<0.1
		Movima	12,213	0.2
		Murato	143	<0.1
		Pacahuara	161	<0.1
		Quechua	1,281,116	18.5
		Sirionó	446	<0.1
		Tacana	11,173	0.2
		Tapiete	99	<0.1
		Tsimane (Chimán)	6,464	0.1
		Weenayek	3,322	<0.1
		Yaminahua	132	<0.1
		Yuki	202	<0.1
		Yuracaré	3,394	<0.1
		Yuracaré-mojeño	292	<0.1
		Others	42,188	0.6
		Not specified	4,419	0.1
		TOTAL	2,794,682	40.4
**Brazil**[1]	2010 Census	Baré	11,990	<0.1
		Guarani Kaiowá	43,401	<0.1
		Kaingang	37,470	<0.1
		Makuxí	28,912	<0.1
		Mundurukú	13,103	<0.1
		Múra	12,479	<0.1
		Pataxó	13,588	<0.1
		Potiguara	20,554	<0.1

		Sateré-Mawé	13,310	<0.1
		Tenetehara	24,428	<0.1
		Terena	28,845	<0.1
		Tikúna	46,045	<0.1
		Xavante	19,259	<0.1
		Xucuru	12,471	<0.1
		Yanomámi	21,982	<0.1
		Monirity groups	470,126	0.2
		TOTAL	817,963	0.4
**Colombia**	2005 Census	Achagua	796	0.1
		Amorua	464	<0.1
		Andoke	136	<0.1
		Arhuaco	21,866	1.5
		Arzario	10,703	0.8
		Awa Kwaiker	25,813	1.9
		Bará	208	<0.1
		Barasana	351	<0.1
		Barí	5,923	0.4
		Betoye	394	<0.1
		Bora	933	0.1
		Calima	76	<0.1
		Cañamomo	21,628	1.6
		Carabayo	26	<0.1
		Carapana	482	<0.1
		Chimila	1,614	0.1
		Chiricoa	46	<0.1
		Chitarero	161	<0.1
		Cocama	2,204	0.2
		Coconuco	16,492	1.2
		Coreguaje	1,767	0.1
		Coyaima	58,810	4.2
		Desano	2,179	0.2
		Dujos	56	<0.1
		Embera	37,327	2.7
		Embera Chamí	29,094	2.1
		Embera katio	38,259	2.8
		Eperara Siapidara	3,853	0.3
		Guambiano	21,085	1.5
		Guanaca	12	<0.1
		Guane	812	0.1
		Guayabero	617	<0.1
		Indigenous from Bolivia	3	<0.1
		Indigenous from Brazil	306	<0.1
		Indigenous from Ecuador	407	<0.1
		Indigenous from Guatemala (Mayas)	7	<0.1
		Indigenous from Mexico	12	<0.1
		Indigenous from Peru	98	<0.1
		Indigenous from Venezuela	8	<0.1
		Inga	15,450	1.1
		Kamëntsa	4,879	0.4
		Kankuama	12,714	0.9
		Karijona	425	<0.1
		Kawiyari	233	<0.1
		Kichwa	481	<0.1
		Kofan	1,657	0.1
		Kogui	9,173	0.7

		Kubeo	3,926	0.3
		Kuiba	769	0.1
		Kurripako	4,340	0.3
		Letuama	202	<0.1
		Makaguaje	145	<0.1
		Makaguane (Hitnu)	656	<0.1
		Makuna	612	<0.1
		Masiguare	268	<0.1
		Matapi	71	<0.1
		Miraña	274	<0.1
		Mokaná	24,825	1.8
		Muisca	14,051	1.0
		Nasa(Páez)	186,178	13.4
		Nonuya	31	<0.1
		Nukak (Maku)	1,348	0.1
		Ocaina	285	<0.1
		Otavaleño	975	0.1
		Panches	8	<0.1
		Piapoco	3,508	0.3
		Piaroa	720	0.1
		Piratapuyo	814	0.1
		Pisamira	151	<0.1
		Puinave	4,178	0.3
		Quillasinga (Pastos)	129,941	9.3
		Quimbaya	163	<0.1
		Sáliba	3,035	0.2
		Senú	233,052	16.7
		Sikuani	19,791	1.4
		Siona	1,829	0.1
		Siriano	544	<0.1
		Tairona	19	<0.1
		Taiwano	166	<0.1
		Tanimuka	342	<0.1
		Tariano	197	<0.1
		Tatuyo	381	<0.1
		Tikuna	7,879	0.6
		Totoro	6,289	0.5
		Tsiripu	17	<0.1
		Tucano	2,016	0.1
		Tule (Kuna)	2,383	0.2
		Tuyuka	444	<0.1
		U´wa (Tunebo)	7,581	0.5
		Uitoto	6,444	0.5
		Wanano	1,305	0.1
		Waunan	9,066	0.7
		Wayuu	270,413	19.4
		Yagua	1,007	0.1
		Yanacona	33,253	2.4
		Yaruro	63	<0.1
		Yauna	99	<0.1
		Yuko	4,761	0.3
		Yukuna	396	<0.1
		Yurutí	377	<0.1
		No information	50,995	3.7
		TOTAL	1,392,623	3.4
**Costa Rica**	2011 Census	Bribi	18,198	0.4

		Brunca or Boruca	5,555	0.1
		Cabécar	16,985	0.4
		Chorotega	11,442	0.3
		Huetar	3,461	0.1
		Maleku or Guatuso	1,780	<0.1
		Nögbe or Guaymí	9,543	0.2
		Terire or Térraba	2,665	0.1
		TOTAL	69,629	1.6
**Ecuador**[2]	2010 Census	A´i Cofán	10,240	<0.1
		Achuar	23,580	<0.1
		Awa	32,202	<0.1
		Chachi	53,605	<0.1
		Chibuleo	37,205	<0.1
		Epera	638	<0.1
		Huaorani	15,047	<0.1
		Kañari	218,648	0.2
		Karanki	38,225	<0.1
		Kayambi	131,811	0.1
		Kichwa Amazonía	108,476	0.1
		Kichwa de Tungurahua	1,924,998	1.7
		Kitu Kara	14,576	<0.1
		Manta Huancavilca Puná	1,972	<0.1
		Natabuela	6,376	<0.1
		Otavalo	307,310	0.3
		Panzaleo	265,436	0.2
		Puruhá	634,394	0.5
		Quisapincha	40,501	<0.1
		Salasaka	51,741	<0.1
		Saraguro	86,543	0.1
		Secoya	2,354	<0.1
		Shiwiar	6,003	<0.1
		Shuar	516,895	0.4
		Siona	2,982	<0.1
		Tsa´chila	14,556	<0.1
		Waranka	5,954	<0.1
		Zapara	3,394	<0.1
		Others indigenous	3,589,747	3.1
		TOTAL	8,145,407	7.0
**Guatemala**	2002 Census	Achi	105,992	0.9
		Akateko	39,370	0.4
		Awakateko	11,068	0.1
		Cho´rti'	46,833	0.4
		Chuj	64,438	0.6
		Itza	1,983	<0.1
		Ixil	95,315	0.8
		Jakalteco	47,024	0.4
		Kaqchikel	832,968	7.4
		K'iche'	1,270,953	11.3
		Mam	617,171	5.5
		Mopan	2,891	<0.1
		Poqomam	42,009	0.4
		Poqomchi'	114,423	1.0
		Q´anjon'al	159,030	1.4
		Q'eqchí'	852,012	7.6
		Sakapulteko	9,763	0.1
		Sipakapense	10,652	0.1

		Tektiteko	2,077	<0.1
		Tzútujil	78,498	0.7
		Uspanteko	7,494	0.1
		Xinka	16,214	0.1
		TOTAL	4,428,178	39.4
**Guyana**[4]	2012 Census	Amerindian	78,492	10.5
**Honduras**	2013 Census	Lenca	453672	5.5
		Maya -Chortí	33256	0.4
		Miskito	80007	1.0
		Nahua	6339	0.1
		Pech	6024	0.1
		Tawahka	2690	<0.1
		Tolupán	19033	0.2
		TOTAL	601,021	7.2
**Mexico**[3]	2010 Census	Aguacateco (Awakateko)	1,920	<0.1
		Amuzgo	1,990	<0.1
		Amuzgo de Guerrero	43,644	<0.1
		Amuzgo de Oaxaca	5,001	<0.1
		Ayapaneco	4	<0.1
		Cakchiquel (Kaqchikel)	143	<0.1
		Chatino	45,019	<0.1
		Chichimeca jonaz	2,190	<0.1
		Chinanteco	131,382	0.1
		Chinanteco de Lalana	1	<0.1
		Chinanteco de Ojitlán	1,938	<0.1
		Chinanteco de Petlapa	9	<0.1
		Chinanteco de Sochiapan	2	<0.1
		Chinanteco de Usila	77	<0.1
		Chinanteco de Valle Nacional	29	<0.1
		Chocho (Chocholteco)	814	<0.1
		Chol (Ch´ol)	212,117	0.2
		Chontal	1,102	<0.1
		Chontal de Oaxaca	4,394	<0.1
		Chontal de Tabasco	36,810	<0.1
		Chuj	2,503	<0.1
		Cochimí	88	<0.1
		Cora	20,078	<0.1
		Cucapá	145	<0.1
		Cuicateco	12,785	<0.1
		Guarijío	2,136	<0.1
		Huasteco	161,120	0.2
		Huave	17,554	<0.1
		Huichol	44,788	<0.1
		Ixcateco	190	<0.1
		Ixil	83	<0.1
		Jacalteco (Jakalteko)	590	<0.1
		Kanjobal (Q´anjob´al)	9,324	<0.1
		Kekchi (Q´eqchi´)	1,248	<0.1
		Kikapú (Kickapoo)	423	<0.1
		Kiliwa	46	<0.1
		Kumiai	289	<0.1
		Lacandón	20	<0.1
		Mame (Mam)	10,374	<0.1
		Matlatzinca	1,096	<0.1
		Maya	786,113	0.8
		Mayo	39,616	<0.1

		Mazahua	135,897	0.1
		Mazateco	223,073	0.2
		Mixe	132,759	0.1
		Mixteco	471,710	0.5
		Mixteco de la costa	27	<0.1
		Mixteco de la mixteca alta	2,493	<0.1
		Mixteco de la mixteca baja	2,197	<0.1
		Mixteco de la zona mazateca	6	<0.1
		Mixteco de Puebla	39	<0.1
		Motocintleco (Qato´k)	106	<0.1
		Náhuatl	1,544,968	1.5
		Ocuilteco (Tlahuica)	737	<0.1
		Otomí	284,992	0.3
		Paipai	199	<0.1
		Pame	11,019	<0.1
		Papabuco	2	<0.1
		Pápago	161	<0.1
		Pima	851	<0.1
		Popoloca	17,964	<0.1
		Popoluca	41,068	<0.1
		Popoluca de la sierra	21	<0.1
		Popoluca de Oluta	1	<0.1
		Popoluca de Texistepec	1	<0.1
		Purépecha (Tarasco)	124,494	0.1
		Quiché (K´iche´)	389	<0.1
		Seri	764	<0.1
		Solteco	10	<0.1
		Tacuate	1,523	<0.1
		Tarahumara	85,018	0.1
		Tepehua	8,868	<0.1
		Tepehuano	2,929	<0.1
		Tepehuano de Chihuahua	7,906	<0.1
		Tepehuano de Durango	25,038	<0.1
		Tlapaneco	120,072	0.1
		Tojolabal	51,733	0.1
		Totonaca (Totonaco)	244,033	0.2
		Triqui	25,883	<0.1
		Tzeltal (Tseltal)	445,856	0.4
		Tzotzil (Tsotsil)	404,704	0.4
		Yaqui	17,116	<0.1
		Zapoteco	425,123	0.4
		Zapoteco de Ixtlán	377	<0.1
		Zapoteco del Istmo	613	<0.1
		Zapoteco del rincón	1	<0.1
		Zapoteco sureño	22,911	<0.1
		Zapoteco vallista	1,394	<0.1
		Zoque	63,022	0.1
		Other indigenous language Mexico	145	<0.1
		Other indigenous language América	833	<0.1
		Indigenous language not specified	144,987	0.1
		TOTAL	6,695,228	6.7
**Nicaragua**	2005 Census	Cacaopera-Matagalpa	15,240	0.3
		Chorotega-Nahua-Mange	46,002	0.9
		Mayangna-Sumu	9,756	0.2

		Miskitu	120,817	2.3
		Nahoa-Nicarao	11,113	0.2
		Rama	4,185	0.1
		Ulwa	698	<0.1
		Xiu-Sutiava	19,949	0.4
		TOTAL	227,760	4.4
**Panama**	2010 Census	Bokota	1,959	<0.1
		Bri Bri	1,068	<0.1
		Buglé	24,912	0.1
		Emberá	31,284	0.1
		Kuna	80,526	0.2
		Ngäbe	260,058	0.8
		Teribe/Naso	4,046	<0.1
		Wounaan	7,279	<0.1
		Other	460	<0.1
		TOTAL	411,592	1.2
**Paraguay**	2012 Census	Ache	1,884	<0.1
	and	Angaité	5,992	0.1
	2012	Ava Guaraní	17,921	0.3
	Indigenous	Ayoreo	2,461	<0.1
	Census	Enlhet Norte	8,167	0.1
		Enxet Sur	7,284	0.1
		Guaná	393	<0.1
		Guaraní Ñandéva	2,470	<0.1
		Guaraní Occidental	3,587	0.1
		Maká	1,888	<0.1
		Manjui	582	<0.1
		Mbya Guaraní	20,546	0.3
		Nivaclé	14,768	0.2
		Paĩ Tavyterã	15,494	0.2
		Qom	1,939	<0.1
		Sanapaná	2,866	<0.1
		Toba Maskoy	2,072	<0.1
		Tomárãho	152	<0.1
		Ybytoso	1,915	<0.1
		Indigenous in the Census 2012	4,769	0.1
		TOTAL	117,150	1.8
**Peru**	2007 Census	Asháninka	67,724	0.3
		Aymará	443,248	1.7
		Quechua	3,360,331	13.0
		Other indigenous language	174,410	0.7
		TOTAL	4,045,713	15.7
**Suriname**[4]	2012 Census	Indigenous	20,344	3.8
**Uruguay**[4]	2011 Census	Indigenous	76,452	2.4

[1] In Brazil exist 305 indigenous groups, but the census report shows only the 15 largest. [2] Information from 2001 census, but proportional to 2010 census. [3] Population of five years or more. [4] The information from Guyana, Suriname and Uruguay is not disaggregated.

Table S14 - Legal standpoint of indigenous people in the countries included in the analyses.

Country	International treaties		Country level[3]		
	ILO 169[1]	UNDRIP[2]	Institution(s) in charge of indigenous affairs	Year of creation	Hierarchical dependence
**Belize**	no	yes	None		
**Bolivia**	yes	yes	Consejo Plurinacional para Vivir Bien en Armonía y Equilibrio con la Madre Tierra	2012	Ministerio de Medio Ambiente y Agua
			Viceministerio de Descolonización	2009	Ministerio de Culturas y Turismo
			Viceministerio de Interculturalidad	2009	Ministerio de Culturas y Turismo
			Viceministerio de Igualdad de Oportunidades	2012	Ministerio de Justicia
			Viceministerio de Autonomías Indígena Originario Campesinas	2009	Ministerio de Autonomías
			Viceministerio de Justicia Indígena Originario Campesina (VJIOC)	2009	Ministerio de Justicia
			Unidad de Políticas Intraculturales, Interculturales y Plurilingüismo (UPIIP)	2010	Ministerio de Educación
			Instituto Plurinacional de Estudio de Lenguas y Culturas	2012	Ministerio de Educación
**Brazil**	yes	yes	Fundação Nacional do Índio (FUNAI)	1967	Ministério da Justiça
**Colombia**	yes	yes	Dirección de Asuntos Indígenas, Minorías y Rom	2005	Ministerio del Interior y de Justicia
			Programa Presidencial para la Formulación de Estrategias y Acciones para el Desarrollo Integral de los Pueblos Indígenas de Colombia	2007	Vicepresidencia de la República
**Costa Rica**	yes	yes	Comisión Nacional de Asuntos Indígenas (CONAI)	1973	Ministerio de Planificación Nacional y Política Económica
**Ecuador**	yes	yes	Consejos Nacionales para la Igualdad	2012	
			Subsecretaría de Pueblos e Interculturalidad	2012	Presidencia del Ecuador, Secretaria de Pueblos, Movimientos Sociales y Participación Ciudadana
**Guatemala**	yes	yes	Fondo de Desarrollo Indígena Guatemalteco (FODIGUA)	1994	Presidencia de la República
			Comisión Presidencial contra la Discriminación y el Racismo contra los Pueblos Indígenas en Guatemala (CODISRA)	1999	Ministerio de Salud Pública y Asistencia Social
			Defensoría de la Mujer Indígena	1999	Secretaría de la Presidencia
**Guyana**[4]	no	yes	Ministry of Indigenous Peoples' Affairs	1992	Office of President
**Honduras**	yes	yes	Secretaría de Estado en los Despachos de Pueblos Indígenas y Afrohondureños (SEDINAFROH)	2010	SEDINAFROH
**Mexico**	yes	yes	Comisión Nacional para el Desarrollo de los Pueblos Indígenas (CDI)	2003	Presidencia de la República
**Nicaragua**	yes	yes	Consejo de Desarrollo de la Costa del Caribe	2007	

**Panama**	no	yes	Dirección Nacional de Política Indígena	2008	Ministerio de Trabajo y Desarrollo Laboral
	yes	yes	Red de Políticas Públicas Indígenas	2012	Ministerio de la Presidencia
**Paraguay**	yes	yes	Instituto Paraguayo del Indígena / Centro de Atención al Indígena (CENADI)	1981	Presidencia de la República
**Peru**	yes	yes	Dirección General de Interculturalidad y Derechos de los Pueblos (incluye al antiguo INDEPA)	2010	Ministerio de Cultura
**Suriname**	no	yes	None		
**Uruguay**	no	yes	None		

[1] Ratification of the International Labour Organization’s Convention No. 169 on Indigenous and Tribal Peoples (1989). [2] Ratification of the United Nations Declaration on the Rights of Indigenous Peoples (2007). [3] Data source: CEPAL, Mujeres indígenas en América Latina. Dinámicas demográficas y sociales en el marco de los derechos humanos. [4] Data source: Technical note on Guyana Indigenous.

Table S15 – P values for comparisons presented in the Figure 2 and Figure 3 of the manuscript.

Coutry/year	Indigenous							
	CPMO		ANC		SBA		DPT	
	Crude	Adjusted	Crude	Adjusted	Crude	Adjusted	Crude	Adjusted
**Belize 2011**	<0.001	<0.001	<0.001	0.002	0.056	0.559	0.813	0.507
**Brazil 2006**	0.438	0.740	0.224	0.223	0.149	0.157	NA	NA
**Bolivia 2008**	<0.001	<0.001	<0.001	<0.001	<0.001	<0.001	0.072	0.059
**Colombia 2010**	<0.001	<0.001	<0.001	<0.001	<0.001	<0.001	0.110	0.708
**Costa Rica 2011**	0.238	0.402	0.033	0.027	0.107	0.119	0.402	0.657
**Ecuador 2004**	<0.001	<0.001	<0.001	<0.001	<0.001	<0.001	0.059	0.371
**Guatemala 2014**	<0.001	<0.001	0.002	0.793	<0.001	<0.001	0.001	0.110
**Guyana 2014**	0.261	0.668	0.001	0.013	<0.001	<0.001	0.217	0.397
**Honduras 2011**	0.077	0.415	0.358	0.754	<0.001	0.029	0.732	0.597
**Mexico 2015**	0.026	0.265	<0.001	0.001	<0.001	<0.001	0.267	0.418
**Nicaragua 2006**	<0.001	0.005	<0.001	0.001	<0.001	0.017	0.019	0.087
**Panama 2013**	<0.001	<0.001	<0.001	0.004	<0.001	<0.001	0.004	0.023
**Paraguay 2008**	0.003	0.041	<0.001	0.118	<0.001	<0.001	0.022	0.420
**Peru 2012**	<0.001	<0.01	<0.001	0.956	<0.001	0.273	0.478	0.724
**Suriname 2010**	0.013	0.288	0.871	0.838	0.099	0.455	<0.000	0.032
	**Afrodescendants**							
**Belize 2011**	0.517	0.207	0.067	0.002	0.516	0.357	0.537	0.183
**Brazil 2006**	0.358	0.489	0.138	0.281	0.056	0.159	NA	NA
**Colombia 2010**	<0.001	<0.001	<0.001	0.218	<0.001	<0.001	0.003	0.033
**Costa Rica 2011**	0.356	0.347	0.216	0.554	0.038	0.066	*	*
**Ecuador 2004**	0.404	0.535	0.582	0.893	0.109	0.105	0.682	0.964
**Guyana 2014**	0.024	0.026	0.546	0.472	0.066	0.196	0.766	0.600

**Honduras 2011**	0.154	0.398	0.001	0.038	0.043	0.546	0.196	0.253
**Panama 2013**	0.929	0.964	0.495	0.818	0.210	0.292	0.093	0.168
**Suriname 2010**	<0.001	<0.001	<0.001	0.014	<0.001	0.968	0.086	0.599
**Uruguay 2012**	NA	NA	0.488	0.763	0.250	0.201	NA	NA

*Sample size < 25. NA: not available.

Table S16 – Intervention coverage by ethnic group (white, brown, black and indigenous), Brazil, 2006.

Indicator/ ethnic group	White	Brown	Black	Indigenous
**Use of modern contraception**	76.6	75.6	72.9	72.5
**Antenatal care (four or more visits)**	92.6	89.4	88.5	82.9
**Skilled attendant at delivery**	98.3	97.4	93.0	91.3
